# CX3CL1/CX3CR1-Mediated Neuron–Microglia Crosstalk Contributes to Methamphetamine-Induced Neuroinflammation and Cognitive Impairment

**DOI:** 10.3390/ijms27198576

**Published:** 2026-09-25

**Authors:** Zhiwen Wang, Genmeng Yang, Ziyan Zhu, Ruike Xu, Xiong Wei, Yunlan Li, Shijun Hong, Yanxia Peng

**Affiliations:** National Health Commission Key Laboratory of Drug Addiction Medicine, School of Forensic Medicine, Kunming Medical University, Kunming 650500, China; wzw4564562022@163.com (Z.W.); yanggenmeng@kmmu.edu.cn (G.Y.); zhuziyan2266@163.com (Z.Z.); xrk0125@126.com (R.X.); wx16687276220@163.com (X.W.); 17384278957@139.com (Y.L.)

**Keywords:** methamphetamine, CX3CL1, CX3CR1, neuroinflammation, microglia, cognitive impairment

## Abstract

Methamphetamine (METH) exposure induces neuroinflammation and cognitive impairment, yet the molecular signals that convert neuronal stress into microglial inflammatory activation remain unclear. This study investigated whether disruption of the CX3CL1/CX3CR1 signaling contributes to METH-induced injury in the prefrontal cortex (PFC). C57BL/6J mice were exposed to METH and assessed for behavioral changes using Y-maze, novel object recognition, and open-field tests. Neuronal injury was evaluated by Nissl staining, and CX3CL1, CX3CR1, and inflammatory mediators were quantified by Western blotting, immunofluorescence, and enzyme-linked immunosorbent assay. Mechanistic validation was performed using neuronal CX3CL1 overexpression and microglial CX3CR1 knockdown models, together with HT-22 neuronal-like cells and BV2 microglial cells in monoculture and coculture systems. METH exposure impaired cognitive performance, increased locomotor activity, induced neuronal injury, and intensified neuroinflammatory signaling. These changes were accompanied by decreased neuronal CX3CL1 and increased microglial CX3CR1 in the PFC, indicating dysregulation of the CX3CL1/CX3CR1 axis. CX3CL1 overexpression partially attenuated METH-induced CX3CR1 upregulation and reduced neuroinflammatory injury, whereas CX3CR1 knockdown suppressed microglial inflammatory activation and improved behavioral outcomes. These findings suggest that CX3CL1/CX3CR1 dysregulation may contribute to METH-induced disruption of neuron–microglia crosstalk and neuroinflammation-associated cognitive impairment.

## 1. Introduction

Methamphetamine (METH) is a highly addictive amphetamine-type psychostimulant with potent central nervous system activity, and its nonmedical use has become a major global public health and forensic concern. Repeated or high-dose exposure can produce persistent neural damage, including neuronal dysfunction, impaired synaptic plasticity, maladaptive neuroimmune activation, and cognitive decline. Evidence from clinical and experimental studies has linked METH-associated neurotoxicity to deficits in learning and memory, executive control, behavioral inhibition, and decision-making. The prefrontal cortex (PFC), which supports working memory, cognitive flexibility, and goal-directed behavior, is considered particularly vulnerable to METH-associated cognitive dysfunction and inflammatory injury. Oxidative stress, monoaminergic disruption, excitotoxic signaling, mitochondrial dysfunction, and neuroinflammation have all been implicated in METH-induced brain injury; however, the intercellular signals that connect neuronal stress, microglial activation, and cognitive impairment remain insufficiently defined [1,2,3,4,5,6,7].

Neuroinflammation is a central pathological feature of METH-induced neurotoxicity and represents a major mechanism through which neural injury may progress to cognitive dysfunction. After METH exposure, damaged or stressed neurons may release multiple danger-associated signals that recruit innate immune pathways within the central nervous system and sustain local production of inflammatory mediators. Microglia, the resident innate immune cells of the brain, are key regulators of these neuroinflammatory responses. Under physiological conditions, microglia dynamically monitor the neural microenvironment and contribute to synaptic pruning, network homeostasis, and tissue repair. Under pathological stimulation, including METH exposure, microglia may shift from a homeostatic surveillance phenotype toward a reactive state characterized by structural remodeling, increased inflammatory mediator release, and enhanced phagocytic and antigen-presenting activity. Persistent microglial activation can amplify the production of proinflammatory cytokines, including TNF-α, IL-1β, and IL-6, thereby creating a feed-forward inflammatory environment that can exacerbate neuronal injury and cognitive dysfunction. Defining the regulatory signals that couple neuronal injury to reactive microglial responses after METH exposure is therefore essential for clarifying the mechanisms underlying METH-associated neuroinflammation and cognitive deficits [4,5,8,9,10,11].

Bidirectional neuron–microglia signaling is required for inflammatory restraint, synaptic regulation, and central nervous system homeostasis. In the context of METH exposure, neurons represent both vulnerable targets of neurotoxicity and active regulators of microglial reactive states through the release or expression of various signaling molecules. Among the molecular systems that coordinate neuron–microglia communication, the CX3CL1/CX3CR1 pathway provides a specialized signaling interface between neuronal and microglial compartments. CX3CL1, or fractalkine, is primarily expressed by neurons in the central nervous system and exists in membrane-bound or soluble forms, whereas CX3CR1 is predominantly enriched on the surface of microglia. Under physiological conditions, neuronal CX3CL1 engagement of microglial CX3CR1 helps maintain microglial homeostasis, modulate synaptic plasticity, and restrain excessive inflammatory activation. In diverse central nervous system disorders, disruption of this pathway has been linked to reactive microglial activation, inflammatory cytokine release, neuronal injury, and cognitive decline. This ligand–receptor axis therefore provides a mechanistic link through which neuronal injury may be coupled to microglial inflammatory responses [12,13,14,15,16].

Despite extensive evidence implicating CX3CL1/CX3CR1 signaling in neurodegeneration, cerebral ischemia, and neuroinflammatory disease, the role of this pathway in METH-induced neurotoxicity remains insufficiently studied. Most existing studies have focused on the direct effects of METH on monoaminergic neurotransmission, oxidative stress, mitochondrial function, and inflammatory cascades. Whether METH disrupts neuron–microglia communication through altered CX3CL1/CX3CR1 signaling, thereby promoting neuroinflammation and cognitive impairment, remains unclear. In particular, the effects of METH on neuronal CX3CL1 expression, microglial CX3CR1 reactivity, and their association with inflammatory phenotypes and PFC-dependent cognitive deficits require further investigation. Direct interventional evidence is also limited regarding whether ligand restoration through CX3CL1 overexpression or receptor suppression through CX3CR1 knockdown can alleviate METH-induced neuroinflammatory injury and cognitive dysfunction. Therefore, exploring METH-induced abnormalities in neuron–microglia neuroimmune crosstalk via the CX3CL1/CX3CR1 axis may help clarify the mechanisms underlying METH-associated neuroinflammatory injury [1,2,3,12,13,14,15,16,17,18,19,20].

The present study examined whether CX3CL1/CX3CR1-mediated neuron–microglia neuroimmune communication contributes to METH-induced neuroinflammation and cognitive impairment. A C57BL/6J mouse model of METH exposure was first established, and the effects of METH on cognitive function and spontaneous locomotor activity were assessed using the Y-maze, novel object recognition, and open-field tests. Neuronal injury, CX3CL1/CX3CR1 pathway alterations, and inflammatory responses in the PFC were evaluated using Nissl staining, Western blot analysis, immunofluorescence, and enzyme-linked immunosorbent assay (ELISA). To test the functional relevance of this pathway in vivo, neuronal CX3CL1 was overexpressed through an adeno-associated virus (AAV)-based strategy, whereas microglial CX3CR1 was selectively knocked down, allowing the functional role of CX3CL1/CX3CR1 signaling to be examined at both the ligand and receptor levels. Complementary monoculture and coculture models using HT-22 neuronal-like cells and BV2 microglial cells were then applied to further examine METH-induced changes in neuron–microglia signaling in vitro. Together, these experiments were designed to determine whether CX3CL1/CX3CR1 dysregulation contributes to METH-induced neuroinflammatory injury and to establish an experimental basis for targeting this pathway in METH-associated neurotoxicity and cognitive impairment.

## 2. Results

### 2.1. METH Exposure Induced Cognitive Impairment, Hyperlocomotor Activity, and Neuronal Injury in the PFC

A mouse model of METH exposure was established to evaluate the effects of repeated METH exposure on cognitive function, spontaneous locomotor behavior, and neuronal injury in the PFC. The experimental procedure is shown in Figure 1A. Mice in the METH group received intraperitoneal injections of METH (10 mg/kg) once daily for 5 consecutive days, whereas mice in the Control group received an equivalent volume of 0.9% saline.

Spatial working memory was evaluated using the Y-maze test. METH-exposed mice showed a significantly lower spontaneous alternation percentage than Control mice, indicating impaired spatial working memory after METH exposure (Figure 1B). Recognition memory was further assessed using the NOR test. In the short-term memory trial, the discrimination index for the novel object was markedly reduced in the METH group compared with the Control group (Figure 1C). This reduction persisted in the long-term memory trial, suggesting sustained impairment of recognition memory after METH exposure (Figure 1D). These behavioral results indicate that repeated METH exposure disrupted spatial working memory and recognition memory in mice.

Spontaneous locomotor behavior was then examined using the open-field test. Locomotor traces were comparable between groups before drug administration. After METH treatment, mice displayed substantially increased movement trajectories, consistent with enhanced spontaneous locomotor activity (Figure 1E). Quantitative analysis showed no significant baseline difference in total distance traveled between the Control and METH groups before administration. In contrast, total distance traveled was significantly increased in the METH group during days 1–5 of treatment and remained elevated during the post-treatment test (Figure 1F). These findings indicate that repeated METH exposure induced a robust hyperlocomotor phenotype.

Nissl staining was performed to determine whether behavioral abnormalities were accompanied by neuronal injury in the PFC. Compared with the Control group, METH-exposed mice showed a significantly lower proportion of morphologically intact Nissl-positive neurons in the PFC, indicating evident neuronal damage after METH exposure (Figure 1G).

Together, these findings show that repeated METH exposure impaired spatial working memory and recognition memory, increased spontaneous locomotor activity, and induced neuronal injury in the PFC. This model therefore provides a basis for subsequent analysis of METH-induced neuroinflammation and disruption of neuron–microglia neuroimmune crosstalk.

### 2.2. METH Exposure Disrupted CX3CL1/CX3CR1 Signaling and Promoted Neuroinflammatory Responses in the PFC

To further determine whether METH exposure altered neuron–microglia signaling in the PFC, CX3CL1/CX3CR1 pathway components and inflammatory markers were examined. Immunofluorescence staining showed that CX3CL1 signals were primarily localized to NeuN-positive neuronal regions in the PFC. Compared with the Control group, METH-exposed mice showed a marked reduction in CX3CL1 fluorescence intensity, indicating diminished neuronal CX3CL1 expression after METH exposure (Figure 2A). Consistently, Western blot analysis confirmed that CX3CL1 protein expression in the PFC was markedly lower in METH-exposed mice than in Control mice (Figure 2C).

CX3CR1 expression was next assessed in relation to the microglial marker Iba1. In contrast to the reduction in CX3CL1, METH exposure significantly increased CX3CR1 fluorescence intensity in the PFC, with CX3CR1 signals largely corresponding to Iba1-positive microglial regions (Figure 2B). Western blot analysis further demonstrated that CX3CR1 protein expression was significantly elevated in the PFC after METH exposure (Figure 2D). These findings indicate that repeated METH exposure was associated with reduced neuronal CX3CL1 and enhanced microglial CX3CR1 expression, consistent with disruption of the CX3CL1/CX3CR1 signaling axis.

Inflammatory phenotypes in the PFC were then evaluated by measuring Arg1, iNOS, IL-1β, and IL-10. Compared with the Control group, METH exposure significantly reduced Arg1 protein expression and increased iNOS protein expression, suggesting a shift toward a proinflammatory state in the PFC (Figure 2E,F). ELISA further showed a parallel cytokine imbalance, with increased IL-1β levels and decreased IL-10 levels in the PFC of METH-exposed mice (Figure 2G,H).

Collectively, these results show that METH exposure altered CX3CL1/CX3CR1 signaling in the PFC, characterized by loss of neuronal CX3CL1 and upregulation of microglial CX3CR1. This signaling imbalance occurred together with increased proinflammatory markers and reduced anti-inflammatory markers, supporting a potential role for abnormal neuron–microglia communication in METH-induced neuroinflammation.

### 2.3. METH Altered CX3CL1/CX3CR1 Signaling and Inflammatory Phenotypes in HT-22 Neuronal-like Cells and BV2 Microglia

To further examine whether METH directly affects neuronal-like cells and microglia, HT-22 cells and BV2 cells were exposed to METH in vitro, and changes in CX3CL1/CX3CR1 signaling, inflammatory mediators, and cell injury were assessed.

CX3CL1 expression was first evaluated in HT-22 neuronal-like cells. Immunofluorescence staining showed a pronounced decrease in CX3CL1 fluorescence intensity after METH treatment compared with the Control group (Figure 3A). Consistently, Western blot analysis demonstrated significantly lower CX3CL1 protein expression in METH-treated HT-22 cells than in Control cells (Figure 3C). These findings indicate that METH exposure suppressed CX3CL1 expression in neuronal-like cells.

CX3CR1 expression was then assessed in BV2 microglial cells. METH treatment markedly increased CX3CR1 fluorescence intensity compared with the Control group (Figure 3B), and Western blot analysis confirmed a significant elevation of CX3CR1 protein expression in METH-treated BV2 cells (Figure 3D). These results indicate that METH exposure induced CX3CR1 upregulation in microglial cells.

To evaluate inflammatory responses after METH treatment, inflammatory mediator release was next examined in culture supernatants. ELISA showed significantly increased IL-1β levels and markedly decreased IL-10 levels after METH treatment (Figure 3E,F), indicating a shift toward a proinflammatory secretory profile. TUNEL staining further revealed a significant increase in the proportion of TUNEL-positive HT-22 neuronal-like cells after METH exposure (Figure 3G), suggesting enhanced apoptosis in neuronal-like cells.

Inflammatory phenotype-related markers were also analyzed in BV2 microglial cells. Immunofluorescence staining revealed that METH treatment significantly reduced Arg1 fluorescence intensity but markedly increased iNOS fluorescence intensity compared with the Control group (Figure 3H,I), suggesting a proinflammatory-like shift in BV2 microglia following drug exposure.

Together, these in vitro findings show that METH exposure decreased CX3CL1 expression in HT-22 neuronal-like cells and increased CX3CR1 expression in BV2 microglial cells, accompanied by neuronal-like cell apoptosis, elevated proinflammatory mediator release, reduced anti-inflammatory cytokine production, and proinflammatory-like microglial activation. These results are consistent with the PFC findings in vivo and support the involvement of disrupted CX3CL1/CX3CR1-mediated neuron–microglia communication in METH-induced neuroinflammatory injury.

### 2.4. CX3CL1 Overexpression Attenuated METH-Induced Behavioral Abnormalities and Neuronal Injury in the PFC

To evaluate the functional role of neuronal CX3CL1 in METH-induced cognitive and behavioral abnormalities, AAV-mediated CX3CL1 overexpression was performed in the PFC before METH exposure. The experimental design is shown in Figure 4A. Mice were assigned to the Control, METH, OE-Control, and OE-METH groups, and behavioral performance and PFC neuronal integrity were then evaluated.

Spatial working memory was assessed using the Y-maze test. METH-exposed mice showed a significant reduction in spontaneous alternation percentage compared with Control mice, confirming impairment of spatial working memory. CX3CL1 overexpression alone did not reduce spontaneous alternation under basal conditions, indicating no detectable adverse effect on spatial working memory. In contrast, spontaneous alternation was significantly higher in the OE-METH group than in the METH group (Figure 4B), indicating that neuronal CX3CL1 overexpression ameliorated METH-induced spatial working memory impairment.

Recognition memory was then evaluated using the NOR test. In both the short-term and long-term memory trials, METH exposure significantly decreased the discrimination index for the novel object compared with the Control group. In contrast, CX3CL1 overexpression markedly increased the discrimination index in METH-exposed mice (Figure 4C), indicating that restoration of CX3CL1 expression improved METH-induced short-term and long-term recognition memory impairment.

The open-field test was performed to evaluate whether CX3CL1 overexpression affected METH-induced hyperlocomotor activity. Locomotor traces showed pronounced increases in movement trajectories during METH treatment, whereas these activity patterns were reduced in the OE-METH group (Figure 4D). Quantitative analysis revealed no significant differences in total distance traveled among groups before drug administration. During days 1–5 of METH treatment and during the post-treatment testing phase, total distance traveled was significantly higher in the METH-exposed group than in the Control group. This increase was significantly reduced in the OE-METH group (Figure 4E), suggesting that CX3CL1 overexpression attenuated METH-induced hyperlocomotor activity.

Nissl staining was then used to assess whether CX3CL1 overexpression protected against PFC neuronal injury. METH exposure significantly decreased the proportion of morphologically intact Nissl-positive neurons in the PFC compared with the Control group. In contrast, the OE-METH group showed a significantly higher proportion of morphologically intact Nissl-positive neurons than the METH group, indicating that CX3CL1 overexpression attenuated METH-induced PFC neuronal injury (Figure 4F). Together, these results demonstrate that restoration of neuronal CX3CL1 expression improved METH-induced spatial working memory and recognition memory impairment, reduced METH-induced hyperlocomotor activity, and preserved neuronal integrity in the PFC. These findings support a protective role for neuronal CX3CL1 signaling in METH-induced behavioral dysfunction and PFC neuronal injury.

### 2.5. CX3CL1 Overexpression Attenuated METH-Induced CX3CR1 Upregulation and Neuroinflammatory Changes in the PFC

The effect of neuronal CX3CL1 overexpression on METH-induced molecular alterations in the PFC was next examined. AAV-CX3CL1 expression was first verified in the target brain region. Fluorescence imaging showed viral expression in the PFC, and Western blot analysis demonstrated significantly higher CX3CL1 protein levels in the OE-CX3CL1 group than in the Control group, confirming successful establishment of the AAV-mediated CX3CL1 overexpression model (Figure 5A).

CX3CL1 expression was then assessed across treatment groups. Immunofluorescence staining showed that CX3CL1 signals were primarily localized to NeuN-positive neuronal regions in the PFC. METH exposure markedly reduced CX3CL1 fluorescence intensity compared with the Control group, whereas CX3CL1 signal was significantly increased in the OE-Control group. In METH-exposed mice, CX3CL1 overexpression restored neuronal CX3CL1 signal, as shown by the markedly higher fluorescence intensity in the OE-METH group than in the METH group (Figure 5B). Western blot analysis produced a consistent pattern: METH exposure significantly decreased CX3CL1 protein expression in the PFC, whereas AAV-mediated CX3CL1 overexpression increased CX3CL1 protein levels under both basal and METH-exposed conditions (Figure 5D).

CX3CR1 expression was further analyzed to determine whether restoration of neuronal CX3CL1 affected the microglial receptor response to METH. Immunofluorescence staining showed significantly increased CX3CR1 fluorescence intensity in the PFC after METH exposure, indicating upregulation of microglial-associated CX3CR1. This increase was reduced in the OE-METH group compared with the METH group, suggesting that CX3CL1 overexpression partially attenuated METH-induced CX3CR1 elevation (Figure 5C). Western blot analysis confirmed that CX3CR1 protein expression was significantly higher in the METH group than in the Control group. CX3CR1 expression was lower in the OE-METH group than in the METH group, although it remained higher than that in the OE-Control group (Figure 5E), indicating partial rather than complete normalization of CX3CR1 expression after CX3CL1 restoration.

Neuroinflammation-related markers were then measured to evaluate whether CX3CL1 overexpression altered the inflammatory response induced by METH. Compared with the Control group, METH exposure significantly increased iNOS protein expression and IL-1β levels while significantly reducing Arg1 protein expression and IL-10 levels in the PFC. In contrast, the OE-METH group showed lower iNOS expression and IL-1β levels, together with higher Arg1 expression and IL-10 levels, compared with the METH group (Figure 5F–I). These results indicate that CX3CL1 overexpression alleviated METH-induced proinflammatory changes and partially restored anti-inflammatory molecular features.

Collectively, these results indicate that AAV-mediated neuronal CX3CL1 overexpression effectively replenished CX3CL1 expression in the PFC and improved METH-induced CX3CR1 upregulation and neuroinflammation-related molecular changes. Together with the behavioral findings, these results suggest that restoration of neuronal CX3CL1 signaling may alleviate METH-induced PFC neuroinflammatory responses and exert protective effects against METH-associated cognitive impairment.

### 2.6. CX3CL1 Overexpression Attenuated METH-Induced Neuronal Injury and Microglial Inflammatory Responses in the Coculture System

To further assess how neuronal CX3CL1 influences METH-induced neuron–microglia interactions, an HT-22/BV2 non-contact coculture system was established. CX3CL1 was overexpressed in HT-22 neuronal-like cells, and subsequent changes in cellular injury, CX3CL1/CX3CR1 signaling, and inflammatory responses were evaluated after METH exposure.

CX3CR1 and CX3CL1 expression were first examined in the coculture system. Immunofluorescence staining showed that METH treatment markedly increased CX3CR1 fluorescence intensity compared with the Control group, indicating enhanced microglial CX3CR1 expression. This increase was reduced in the OE-METH group, suggesting that neuronal CX3CL1 overexpression attenuated METH-induced CX3CR1 upregulation in BV2 microglia (Figure 6A). Conversely, CX3CL1 fluorescence intensity decreased after METH treatment but increased substantially in the OE-Control group. CX3CL1 fluorescence intensity was higher in the OE-METH group than in the METH group, indicating partial restoration of CX3CL1 expression through neuronal CX3CL1 overexpression (Figure 6B).

Western blot analysis further supported the immunofluorescence findings. CX3CL1 protein expression was significantly increased in the OE-Control group compared with the Control group, indicating efficient CX3CL1 overexpression in the coculture system (Figure 6C). METH treatment reduced CX3CL1 protein expression, whereas CX3CL1 overexpression increased CX3CL1 levels under METH exposure (Figure 6G). In parallel, METH increased CX3CR1 protein expression, whereas CX3CR1 levels were lower in the OE-METH group than in the METH group, further indicating that neuronal CX3CL1 overexpression partially attenuated METH-induced CX3CR1 upregulation (Figure 6F).

Inflammatory mediator release was then evaluated by measuring IL-1β and IL-10 levels in culture supernatants. METH treatment significantly increased IL-1β levels and decreased IL-10 levels compared with the Control group, indicating a proinflammatory shift in the coculture environment. Compared with the METH group, the OE-METH group showed reduced IL-1β levels and increased IL-10 levels, suggesting that neuronal CX3CL1 overexpression mitigated METH-induced proinflammatory signaling and partially restored anti-inflammatory cytokine production (Figure 6D,E).

TUNEL staining was further performed to assess injury in HT-22 neuronal-like cells. METH treatment significantly increased the proportion of TUNEL-positive cells, indicating enhanced apoptosis. This increase was markedly reduced in the OE-METH group, suggesting that CX3CL1 overexpression alleviated METH-induced apoptosis in HT-22 neuronal-like cells within the coculture system (Figure 6H).

Inflammatory phenotype-related markers in BV2 microglial cells were also examined. METH treatment increased iNOS fluorescence intensity and decreased Arg1 fluorescence intensity compared with the Control group, consistent with a proinflammatory-like microglial shift. In contrast, the OE-METH group showed decreased iNOS expression and increased Arg1 expression compared with the METH group, indicating that neuronal CX3CL1 overexpression suppressed METH-induced proinflammatory-like changes in BV2 microglia and partially restored anti-inflammatory features (Figure 6I,J).

Together, these results show that METH exposure disrupted CX3CL1/CX3CR1 signaling in the HT-22/BV2 coculture system, with reduced neuronal CX3CL1 expression, increased microglial CX3CR1 expression, enhanced neuronal-like cell apoptosis, and amplified inflammatory responses. Neuronal CX3CL1 overexpression alleviated these abnormalities, further supporting a protective role for neuronal CX3CL1 signaling in limiting METH-induced inflammatory neuron–microglia interactions.

### 2.7. CX3CR1 Knockdown Attenuated METH-Induced Behavioral Abnormalities and Neuronal Injury in the PFC

To further evaluate the role of microglial CX3CR1 in METH-induced cognitive impairment, hyperlocomotor activity, and PFC neuronal injury, AAV-mediated CX3CR1 knockdown was performed before METH exposure. The experimental design is shown in Figure 7A. Mice were assigned to the Control, METH, KD-Control, and KD-METH groups, followed by behavioral testing and assessment of neuronal integrity in the PFC.

Spatial working memory was assessed using the Y-maze test. Compared with the Control group, METH-exposed mice showed a significant reduction in spontaneous alternation percentage, indicating impaired spatial working memory after METH exposure. CX3CR1 knockdown alone did not reduce spontaneous alternation under basal conditions, suggesting no detectable adverse effect on spatial working memory. In contrast, spontaneous alternation was significantly higher in the KD-METH group than in the METH group, indicating that CX3CR1 knockdown improved METH-induced spatial working memory impairment (Figure 7B).

Recognition memory was then evaluated using the NOR test. In both the short-term and long-term memory trials, METH exposure significantly reduced the discrimination index compared with the Control group. The KD-METH group showed a markedly higher discrimination index than the METH group at both time points, indicating that CX3CR1 knockdown improved METH-induced short-term and long-term recognition memory impairment. No obvious reduction in the discrimination index was observed in the KD-Control group compared with the Control group, suggesting that CX3CR1 knockdown did not markedly impair recognition memory under basal conditions (Figure 7C).

The open-field test was further performed to determine whether CX3CR1 knockdown affected METH-induced hyperlocomotor activity. Locomotor traces showed substantially increased movement trajectories in the METH group during METH treatment, whereas activity traces were reduced in the KD-METH group compared with the METH group (Figure 7D). Quantitative analysis showed no significant difference in total distance traveled among groups before drug administration. During days 1–5 of METH treatment and the post-treatment testing phase, total distance traveled was significantly higher in the METH group than in the Control group, confirming METH-induced hyperlocomotor activity. This increase was significantly reduced in the KD-METH group, suggesting that CX3CR1 knockdown attenuated METH-induced hyperlocomotor activity (Figure 7E).

Nissl staining was further applied to assess the effect of CX3CR1 knockdown on METH-induced PFC neuronal injury. METH exposure significantly decreased the proportion of morphologically intact Nissl-positive neurons in the PFC compared with the Control group. CX3CR1 knockdown alone did not reduce neuronal integrity under basal conditions. In METH-exposed mice, however, CX3CR1 knockdown markedly increased the proportion of morphologically intact Nissl-positive neurons compared with the METH group, indicating reduced PFC neuronal injury (Figure 7F).

Collectively, these results indicate that CX3CR1 knockdown improved METH-induced spatial working memory and recognition memory deficits, reduced METH-induced hyperlocomotor activity, and alleviated PFC neuronal injury. These findings suggest that microglial CX3CR1 upregulation may participate in METH-induced behavioral abnormalities and neuronal injury, whereas suppressing CX3CR1 signaling may provide neurobehavioral protection after METH exposure.

### 2.8. CX3CR1 Knockdown Attenuated METH-Induced Neuroinflammation-Related Changes in the PFC

The effect of CX3CR1 knockdown on METH-induced molecular abnormalities in the PFC was next examined. AAV-mediated CX3CR1 knockdown efficiency was first validated in the target brain region. Reporter fluorescence signals showed viral expression in the PFC, and Western blot analysis demonstrated significantly lower CX3CR1 protein levels in the KD-CX3CR1 group than in the Control group, confirming successful establishment of the AAV-mediated CX3CR1 knockdown model (Figure 8A).

Neuronal CX3CL1 expression was then assessed across groups. Immunofluorescence staining showed that CX3CL1 signals were mainly localized to NeuN-positive neuronal regions in the PFC. METH exposure markedly reduced CX3CL1 fluorescence intensity compared with the Control group. This reduction was partially reversed in the KD-METH group, as shown by higher CX3CL1 fluorescence intensity than in the METH group (Figure 8B). Western blot analysis showed a consistent pattern: METH decreased CX3CL1 protein expression in the PFC, whereas CX3CR1 knockdown partially restored CX3CL1 protein levels under METH exposure (Figure 8D).

CX3CR1 expression was further examined to determine whether knockdown limited METH-induced receptor upregulation. Immunofluorescence staining showed significantly increased CX3CR1 fluorescence intensity in the PFC after METH exposure, consistent with enhanced microglial-associated CX3CR1 expression. Compared with the METH group, the KD-METH group showed markedly reduced CX3CR1 fluorescence intensity, indicating suppression of METH-induced CX3CR1 upregulation (Figure 8C). Western blot analysis further showed that METH increased CX3CR1 protein expression in the PFC, whereas AAV-shCX3CR1 reduced this METH-induced upregulation (Figure 8E). These results indicate that CX3CR1 knockdown effectively reduced abnormal receptor-side CX3CR1 expression in the PFC after METH exposure.

Neuroinflammation-related markers were then measured to determine whether CX3CR1 knockdown modified the inflammatory phenotype induced by METH. Western blot analysis showed that METH significantly increased iNOS protein expression and decreased Arg1 protein expression in the PFC compared with the Control group. In contrast, the KD-METH group showed lower iNOS expression and higher Arg1 expression than the METH group, indicating that CX3CR1 knockdown alleviated METH-induced proinflammatory-like phenotypic changes (Figure 8F,G).

Cytokine measurements further supported this anti-inflammatory effect. ELISA showed that METH exposure increased IL-1β levels and decreased IL-10 levels in the PFC compared with the Control group. Compared with the METH group, the KD-METH group showed reduced IL-1β levels and increased IL-10 levels (Figure 8H,I), indicating that CX3CR1 knockdown suppressed proinflammatory cytokine production and partially restored anti-inflammatory cytokine expression after METH exposure.

Together, these findings show that CX3CR1 knockdown reduced METH-induced CX3CR1 upregulation in the PFC, partially reversed the decrease in CX3CL1 expression, decreased iNOS and IL-1β levels, and increased Arg1 and IL-10 levels. Combined with the behavioral and Nissl staining results, these data suggest that microglial CX3CR1 participates in METH-induced PFC neuroinflammation and neuronal injury, and that suppressing CX3CR1 signaling may alleviate CX3CL1/CX3CR1 axis dysregulation and associated inflammatory responses at the receptor level.

### 2.9. CX3CR1 Knockdown Attenuated METH-Induced Neuronal Injury and Microglial Inflammatory Responses in the Coculture System

To further define the role of microglial CX3CR1 in METH-induced neuron–microglia interactions, an HT-22/BV2 coculture system was established in which CX3CR1 was knocked down in BV2 microglial cells. This model was used to evaluate whether reducing microglial CX3CR1 expression could modify METH-induced cellular injury, CX3CL1/CX3CR1 signaling imbalance, and inflammatory activation.

The expression levels of CX3CR1 and CX3CL1 were first examined in the coculture system. Immunofluorescence staining revealed that METH treatment markedly increased CX3CR1 fluorescence intensity compared with the Control group, indicating enhanced microglial CX3CR1 expression. This increase was reduced in the KD-METH group, demonstrating that CX3CR1 knockdown attenuated METH-induced CX3CR1 upregulation (Figure 9A). In parallel, METH treatment decreased CX3CL1 fluorescence intensity, whereas the KD-METH group showed higher CX3CL1 fluorescence intensity than the METH group, indicating that CX3CR1 knockdown partially reversed the METH-induced reduction in neuronal CX3CL1 expression (Figure 9B).

Western blot analysis further verified the efficiency of CX3CR1 knockdown and associated molecular changes. CX3CR1 protein expression was significantly lower in the KD-Control group than in the Control group, confirming efficient CX3CR1 suppression in the coculture model (Figure 9C). METH treatment reduced CX3CL1 protein expression, whereas CX3CL1 levels were partially increased in the KD-METH group compared with the METH group (Figure 9F). Conversely, METH increased CX3CR1 protein expression, whereas CX3CR1 protein expression was lower in the KD-METH group than in the METH group, further indicating that CX3CR1 knockdown effectively suppressed METH-induced abnormal receptor-side expression (Figure 9G).

Inflammatory mediator release was then assessed by measuring IL-1β and IL-10 levels in culture supernatants. METH treatment significantly increased IL-1β levels and decreased IL-10 levels compared with the Control group, indicating a shift toward a proinflammatory secretory profile. Compared with the METH group, the KD-METH group showed lower IL-1β levels and higher IL-10 levels, suggesting that CX3CR1 knockdown reduced METH-induced proinflammatory signaling and partially restored anti-inflammatory cytokine production (Figure 9D,E).

TUNEL staining was further performed to evaluate injury in HT-22 neuronal-like cells. METH treatment significantly increased the proportion of TUNEL-positive cells, indicating enhanced apoptosis. This increase was markedly reduced in the KD-METH group, suggesting that CX3CR1 knockdown alleviated METH-induced apoptosis in HT-22 neuronal-like cells within the coculture system (Figure 9H).

Inflammatory phenotype-related markers in BV2 microglial cells were also examined. Immunofluorescence staining revealed that METH treatment increased iNOS fluorescence intensity and decreased Arg1 fluorescence intensity compared with the Control group, consistent with a proinflammatory-like microglial shift. In contrast, the KD-METH group showed decreased iNOS expression and increased Arg1 expression compared with the METH group, indicating that CX3CR1 knockdown suppressed METH-induced proinflammatory-like changes in BV2 microglia and partially restored anti-inflammatory features (Figure 9I,J).

Collectively, these results show that METH treatment disrupted CX3CL1/CX3CR1 signaling in the HT-22/BV2 coculture system, with increased microglial CX3CR1 expression, reduced neuronal CX3CL1 expression, enhanced neuronal-like cell apoptosis, and intensified inflammatory responses. CX3CR1 knockdown alleviated these abnormalities, supporting the involvement of microglial CX3CR1 in METH-induced inflammatory neuron–microglia interactions and suggesting that CX3CR1 inhibition may reduce METH-induced neuronal injury and inflammatory activation.

## 3. Discussion

This study identified CX3CL1/CX3CR1 signaling as a neuroimmune interface associated with METH-induced PFC neuronal injury, inflammatory activation, cognitive impairment, and hyperlocomotor activity in mice. Repeated METH exposure impaired spatial working memory in the Y-maze test and recognition memory in the NOR test, while increasing locomotor activity in the open-field test. These behavioral abnormalities were accompanied by a reduction in morphologically intact Nissl-positive neurons in the PFC, indicating neuronal injury in a brain region critical for executive control and cognitive flexibility. METH exposure also shifted the PFC inflammatory profile toward a proinflammatory state, as reflected by increased iNOS and IL-1β levels and decreased Arg1 and IL-10 levels. Consistent with the in vivo findings, METH increased apoptosis in HT-22 neuronal-like cells and promoted proinflammatory marker changes in BV2 microglial cells, supporting a close relationship between METH-induced neuronal injury and protective effects. SHT-22/BV2d microglial inflammatory activation. These findings suggest that METH-induced PFC dysfunction does not arise from isolated injury within a single cellular compartment but may involve reciprocal interactions between neuronal damage and microglial inflammatory responses. Neurons are primary targets of METH neurotoxicity, whereas microglia are principal immune effector cells in the central nervous system. Neuronal injury can disrupt the signals that normally regulate microglial activity, and sustained microglial reactivity can, in turn, release inflammatory mediators that further damage neurons and amplify cognitive dysfunction. This reciprocal process provides a biologically plausible framework for understanding METH-induced PFC neuroinflammation and cognitive impairment beyond mechanisms centered only on individual inflammatory mediators or isolated cell-type responses. Within this framework, the present study showed that METH exposure reduced neuronal-associated CX3CL1 expression and increased microglial-associated CX3CR1 expression in the PFC, indicating disruption of CX3CL1/CX3CR1-mediated neuron–microglia signaling. Functional manipulation of this pathway further supported its relevance to METH-induced pathology. AAV-mediated CX3CL1 overexpression restored ligand-side signaling and alleviated neuroinflammation, neuronal injury, and behavioral abnormalities after METH exposure. Conversely, AAV-mediated CX3CR1 knockdown reduced receptor-side CX3CR1 upregulation and produced similar coculture experiments further demonstrated that manipulation of either neuronal CX3CL1 or microglial CX3CR1 mitigated METH-induced inflammatory neuron–microglia interactions in vitro. Together, these results define the CX3CL1/CX3CR1 axis as part of a broader neuroimmune communication mechanism underlying METH-induced PFC injury. Rather than representing a secondary inflammatory response alone, METH-induced neuroinflammation may involve abnormal coupling between neuronal injury signals and microglial immune activation.

Neuronal CX3CL1 overexpression further indicated that the METH-induced decline in CX3CL1 was unlikely to be a passive molecular change. Instead, reduced neuronal CX3CL1 signaling may contribute to the inflammatory and neurotoxic cascade triggered by METH. In the present study, AAV-mediated CX3CL1 overexpression improved Y-maze and NOR performance, reduced hyperlocomotor activity in the open-field test, and attenuated neuronal injury in the PFC after METH exposure. Restoration of neuronal CX3CL1 signaling also shifted the inflammatory profile toward a less reactive state, with decreased iNOS and IL-1β levels and increased Arg1 and IL-10 levels. In the HT-22/BV2 coculture system, CX3CL1 overexpression in HT-22 neuronal-like cells similarly reduced METH-induced proinflammatory changes in BV2 microglia and decreased apoptosis in HT-22 neuronal-like cells, supporting a role for neuronal CX3CL1 in constraining microglial inflammatory activation. Notably, CX3CL1 overexpression partially attenuated METH-induced CX3CR1 upregulation, although this effect should not be interpreted as direct suppression of CX3CR1 expression by CX3CL1. The protective effects of CX3CL1 restoration likely reflect both reconstitution of neuronal ligand signaling and secondary dampening of microglial reactivity after reduction of neuronal injury and inflammatory stress. Thus, CX3CL1 overexpression is more appropriately interpreted as a means of restoring dysregulated CX3CL1/CX3CR1-mediated neuron–microglia communication, rather than as protection solely through CX3CR1 downregulation.

CX3CR1 knockdown further supported the role of this axis in METH neurotoxicity from the microglial receptor side. METH exposure markedly increased CX3CR1 expression in the PFC and in BV2 microglial cells, in parallel with enhanced proinflammatory markers. Suppression of CX3CR1 improved METH-induced cognitive impairment, hyperlocomotor activity, and PFC neuronal injury, while reducing iNOS and IL-1β levels and increasing Arg1 and IL-10 levels. In the coculture system, CX3CR1 knockdown in BV2 microglia also decreased METH-induced apoptosis in HT-22 neuronal-like cells and mitigated abnormal cytokine changes. These findings suggest that microglial CX3CR1 upregulation contributes to METH-induced proinflammatory responses and neuronal injury and that suppressing CX3CR1 expression may weaken inflammatory neuron–microglia crosstalk from the receptor side. Compared with CX3CL1 overexpression, CX3CR1 knockdown provides more direct evidence for a pathological role of microglial CX3CR1 in METH-induced neuroinflammation, although the contribution of additional inflammatory pathways and intercellular signals cannot be excluded. Thus, the CX3CL1/CX3CR1 axis represents an important regulatory node in METH-induced neuroimmune crosstalk, rather than the sole mechanism driving this process.

Several limitations should be acknowledged. First, the present findings support the involvement of the CX3CL1/CX3CR1 axis in METH-induced neuroinflammation and cognitive impairment based on coordinated expression changes and functional manipulation, but the intracellular signaling events associated with CX3CR1 activation were not directly examined. iNOS and Arg1 were used as inflammation- and microglial phenotype-related markers rather than as direct downstream effectors of CX3CR1. Moreover, the microglial phenotype was characterized using a limited panel of inflammation-related markers, primarily iNOS, Arg1, IL-1β, and IL-10, and should therefore be interpreted as reflecting proinflammatory-like changes rather than a comprehensive microglial activation state. Additional microglial markers, morphological analyses, and broader inflammatory mediator profiling were not performed. Furthermore, although CX3CR1 immunofluorescence was assessed in relation to Iba1-positive microglial regions, CX3CR1 intensity was not normalized on a per-Iba1-positive-cell basis and Iba1-positive cell density was not independently quantified. Thus, the present data cannot distinguish unequivocally between increased CX3CR1 expression per microglial cell and changes associated with microglial abundance or reactive state. Accordingly, the inflammatory responses observed after METH exposure cannot be fully attributed to CX3CR1-dependent signaling, as additional inflammatory pathways and intercellular signals are likely to contribute. Second, although CX3CL1 exists in both membrane-bound and soluble forms, the present study did not specifically distinguish or quantify these two forms. In addition, the non-contact Transwell coculture system does not directly assess contact-dependent signaling mediated by membrane-bound CX3CL1. Third, the analysis primarily focused on the PFC, although METH-induced neurotoxicity can affect multiple brain regions, including the hippocampus and striatum. CX3CL1/CX3CR1 signaling was not examined in these regions, and therefore the regional molecular conclusions of the present study are restricted to the PFC. Fourth, the HT-22/BV2 coculture system cannot fully recapitulate the complexity of the PFC in vivo. Moreover, the 2 mmol/L METH exposure used in vitro represents a relatively strong toxicological challenge, and nonspecific cytotoxic effects cannot be fully excluded. This condition was based on previous concentration- and time-response studies in HT-22 cells and was not independently optimized in BV2 cells. In vivo, brain or plasma METH concentrations and core body temperature were not measured, and welfare-monitoring parameters were not analyzed as quantitative outcomes. Therefore, systemic physiological effects of METH may have contributed to the observed phenotypes and cannot be fully separated from CX3CL1/CX3CR1-related effects. Finally, the present study primarily assessed endpoint changes after METH exposure, and time-course analyses were not performed. In addition, because only male C57BL/6J mice were used, the generalizability of these findings to females, other strains or species, and humans remains to be determined.

In summary, this study provides evidence that dysregulated CX3CL1/CX3CR1 signaling contributes to METH-induced PFC neuroinflammation and cognitive impairment within a neuron–microglia neuroimmune crosstalk framework. METH exposure reduced neuronal CX3CL1 expression and increased microglial CX3CR1 expression, accompanied by proinflammatory activation, neuronal injury, and behavioral abnormalities. Restoration of neuronal CX3CL1 or knockdown of microglial CX3CR1 partially alleviated these pathological changes. These findings indicate that dysregulation of the CX3CL1/CX3CR1 axis represents an important neuroimmune signaling node that links neuronal injury to microglial inflammatory responses after METH exposure and may serve as a potential target for intervention in METH-associated neurotoxicity and cognitive impairment.

## 4. Materials and Methods

### 4.1. Animal Experiments

(1) Male C57BL/6J mice aged 6–8 weeks and weighing 21–30 g were obtained from SPF (Beijing) Biotechnology Co., Ltd. (Beijing, China; production license No. SCXK (Jing) 2024-0001). Mice were housed under specific-pathogen-free conditions at 22 ± 1 °C and 50 ± 10% relative humidity, with a 12 h light/12 h dark cycle and free access to food and water. Mice were acclimatized to the animal facility for 7 days before the experiments. All animal procedures were conducted in the laboratory animal facility of Kunming Medical University. All procedures were conducted in accordance with relevant regulations for laboratory animal welfare and were approved by the Animal Ethics Committee of Kunming Medical University (protocol code Kmmu20230301; approved on 17 February 2023).

(2) For the METH exposure experiment, mice were randomly assigned to the Control group, which received saline intraperitoneally (10 mL/kg) or the METH group, which received METH intraperitoneally (10 mg/kg). The METH regimen used in the present study (10 mg/kg, i.p., once daily for 5 consecutive days) was selected with reference to previously published evidence showing that repeated 10 mg/kg METH exposure over 5 consecutive days induces spatial learning and memory impairment in C57BL/6 mice [21], together with preliminary experiments performed in our laboratory. For CX3CL1 overexpression (OE) or CX3CR1 knockdown (KD) in the PFC via stereotaxic injection, mice were randomly assigned to the Control, METH, OE-CX3CL1 + Control, OE-CX3CL1 + METH, KD-CX3CR1 + Control, and KD-CX3CR1 + METH groups using a random-number table. Drug administration, behavioral testing, and tissue collection were performed in an interleaved order across groups to minimize order-related confounding. After testing, mice were deeply anesthetized with isoflurane. For biochemical assays, mice were euthanized by decapitation, and the PFC was rapidly dissected on ice and stored at −80 °C. For histological and immunofluorescence analyses, mice were transcardially perfused with PBS followed by 4% paraformaldehyde, and the PFC was collected for fixation and staining. Twelve mice were initially allocated to each group. The basic METH exposure experiment included two groups and 24 mice; the CX3CL1-overexpression experiment included four groups and 48 mice; and the CX3CR1-knockdown experiment included four groups and 48 mice. A total of 120 mice were used. No formal a priori sample size calculation was performed. The sample size of 12 mice per group was determined based on previous comparable METH studies, preliminary experimental results, and laboratory experience, while ensuring sufficient animals for behavioral, histological, and biochemical analyses. No formal inclusion or exclusion criteria were established a priori. No animals were excluded on the basis of predefined experimental criteria, and no data obtained from animals selected for a given analysis were subsequently excluded from that analysis. Different analytical endpoints used prespecified subsets of the 12 mice allocated to each group. Behavioral analyses included 6 mice per group, Nissl staining included 3 mice per group, immunofluorescence included 4 mice per group, Western blot analysis included 3 mice per group, and ELISA included 5 mice per group. Histological and immunofluorescence analyses required transcardial perfusion and fixation of brain tissue, whereas Western blot and ELISA analyses required fresh, non-fixed PFC tissue; therefore, these two terminal tissue-processing routes were mutually exclusive. Within the same tissue-processing route, animals could contribute to more than one compatible analytical endpoint. Behavioral testing was performed before terminal tissue collection. Exact sample sizes for each analysis are also reported in the corresponding figure legends.

(3) AAV-based gene delivery was used to manipulate CX3CL1 and CX3CR1 expression in the PFC. The AAV vectors were commercially constructed and packaged by Hanbio Biotechnology Co., Ltd. (Shanghai, China). Mice assigned to the overexpression group received AAV-CX3CL1, whereas mice assigned to the knockdown group received AAV-shCX3CR1. After isoflurane anesthesia, mice were secured in a stereotaxic apparatus (Model 68025; RWD Life Science Co., Ltd., Shenzhen, China), with bregma used as the reference point. AAV was bilaterally injected into the PFC at AP +2.4 mm and ML ±0.3 mm. At each hemisphere, 300 nL of viral suspension was injected at each of two dorsoventral depths (DV −2.8 and −2.0 mm) at approximately 60 nL/min. The needle was retained for 5–8 min after each injection to minimize reflux before slow withdrawal. The scalp was sutured after injection. Mice were monitored daily for body weight, general appearance, general activity, food and water intake, and surgical wound condition during the recovery and subsequent experimental periods. These parameters were used for routine animal-welfare and humane-endpoint monitoring rather than as prespecified quantitative study outcomes and were not subjected to statistical analysis. Core body temperature was not quantitatively recorded. Humane endpoints included a body-weight loss of more than 20%, persistent inability to eat or drink, severe lethargy, or serious wound infection. No unexpected adverse events or procedure-related deaths occurred during the study. Mice were allowed to recover for 3–4 weeks before subsequent experiments. CX3CL1 overexpression and CX3CR1 knockdown efficiencies were verified by Western blot analysis.

(4) Behavioral tests

Behavioral tests were used to assess locomotor activity (open-field test), spatial working memory (Y-maze test), and recognition memory using the novel object recognition (NOR) test.

Behavioral tests were recorded and analyzed using VisuTrack software (V2.0; XR-VT; Shanghai Xinruan Information Technology Co., Ltd., Shanghai, China).

A. In the open-field test, each mouse was gently placed in the center of the open-field arena and allowed to explore freely. Locomotor activity was recorded using a video-tracking system, and total distance traveled was used as the primary outcome measure. Testing was conducted before dosing, on days 1–5 of METH treatment, and on day 6 after treatment ended. The arena was cleaned with 75% ethanol between trials.

B. The Y-maze apparatus consisted of three equal-length arms positioned at 120° angles. Each mouse was placed at the end of one arm and allowed to explore freely for 5 min. The sequence of arm entries was recorded, and entry into three different arms consecutively was defined as a correct spontaneous alternation, using overlapping counts. Spontaneous alternation was calculated as follows: spontaneous alternation (%) = correct alternations/(total arm entries − 2) × 100%. The apparatus was cleaned with 75% ethanol after each trial.

C. The NOR test included habituation, training, and testing phases. During training, two identical objects were placed in the arena, and each mouse was allowed to explore freely for 5 min. Testing was performed 2 h and 24 h after training to assess short-term and long-term memory, respectively. During each test phase, one familiar object was replaced by a novel object, and exploration times for the novel and familiar objects were recorded. The discrimination index was calculated as follows: discrimination index = (novel object exploration time − familiar object exploration time)/(novel object exploration time + familiar object exploration time). The arena and objects were cleaned with 75% ethanol after each trial.

### 4.2. Cell Experiments

BV2 mouse microglial cells (Cat. No. SCSP-5208) and HT-22 mouse hippocampal neuronal cells (Cat. No. SCSP-5419) were obtained from the Cell Bank of the Chinese Academy of Sciences (Shanghai, China).

(1) BV2 mouse microglial cells were cultured in Dulbecco’s Modified Eagle Medium (DMEM) containing 10% fetal bovine serum (FBS) and 1% penicillin–streptomycin at 37 °C in a humidified atmosphere containing 5% CO_2_. Cells were seeded in 6-well plates at 2 × 10^5^–3 × 10^5^ cells/well. After reaching the logarithmic growth phase, the culture medium was replaced with serum-free DMEM, and cells were exposed to 2 mmol/L METH for 24 h to induce an in vitro microglial inflammatory response. Cells and culture supernatants were then collected for downstream analyses according to the experimental design.

(2) HT-22 mouse hippocampal neuronal cells were cultured in DMEM containing 10% FBS and 1% penicillin–streptomycin at 37 °C and 5% CO_2_. Cells were seeded in 6-well plates at 2 × 10^5^–3 × 10^5^ cells/well. After reaching the logarithmic growth phase, the culture medium was replaced with serum-free DMEM, and cells were exposed to 2 mmol/L METH for 24 h to establish an in vitro neurotoxicity model. Treated cells were collected for subsequent assays. For HT-22 cells, the METH exposure condition of 2 mmol/L for 24 h was selected based on previously established concentration- and time-response studies from our laboratory [22,23]. The same METH exposure condition was used for BV2 cells to maintain a consistent exposure condition for both cell types in the coculture system.

(3) A non-contact Transwell coculture system was established using 6-well Transwell inserts with 0.4-μm pores. HT-22 cells were seeded in the lower chamber at 2 × 10^5^ cells/well in 2 mL complete medium and cultured overnight. On the following day, BV2 cells were seeded in the upper inserts at 1 × 10^5^ cells/insert in 1 mL medium, and the inserts were placed into the wells containing HT-22 cells. After assembly of the coculture system, the medium in both compartments was replaced with serum-free DMEM, and cells were exposed to 2 mmol/L METH for 24 h. Cells from both compartments and culture supernatants were collected according to the downstream assays.

(4) Stable CX3CL1-overexpressing HT-22 cells and CX3CR1-knockdown BV2 cells were generated by lentiviral transduction. Lentiviral vectors and their corresponding control vectors were commercially constructed and packaged by Hanbio Biotechnology (Shanghai, China). HT-22 and BV2 cells were seeded in 24-well plates at 1 × 10^5^ cells/well and transduced 24 h after seeding, after cell attachment. Lentiviral amounts were calculated on the basis of the initial seeding number to achieve an MOI of 4. HT-22 cells were transduced with HBLV-m-Cx3cl1-3xflag-ZsGreen-PURO (3 × 10^8^ TU/mL; Hanbio product code LV109111226). HBLV-ZsGreen-PURO overexpression-control lentivirus (2 × 10^8^ TU/mL; Hanbio product code LV109111225), containing the corresponding vector backbone without the Cx3cl1 overexpression insert, was used as the control. BV2 cells were transduced with HBLV-m-Cx3cr1 shRNA1-ZsGreen-PURO (3.5 × 10^8^ TU/mL; Hanbio product code LV114040101). The sense-strand target sequence of CX3CR1 shRNA1 was 5′-CCAAGAGCATCACTGACAT-3′. HBLV-ZsGreen-PURO NC lentivirus (3 × 10^8^ TU/mL; Hanbio product code LV111010110), carrying the non-targeting scrambled sequence 5′-TTCTCCGAACGTGTCACGTAA-3′, was used as the negative control. The negative-control construct used the corresponding vector backbone and was packaged under the same conditions as the experimental lentivirus. Polybrene (hexadimethrine bromide) was added at a final concentration of 5 μg/mL during transduction. After 24 h of viral exposure, the virus-containing medium was replaced with fresh complete DMEM containing 10% FBS and 1% penicillin–streptomycin. Puromycin selection was initiated 48 h after lentiviral transduction. For BV2 cells, the working concentration of puromycin was determined using a preliminary kill-curve assay; 2 μg/mL reduced the viability of non-transduced BV2 cells to near-background levels and was therefore selected for stable-cell screening. For HT-22 cells, 2 μg/mL puromycin was selected based on a previously established and published HT-22 lentiviral-selection protocol from our laboratory [23]. Puromycin selection was continued for 7 days, with the selection medium replaced every 2–3 days. CX3CL1 overexpression in HT-22 cells and CX3CR1 knockdown in BV2 cells were assessed by Western blot analysis before subsequent experiments.

### 4.3. Western Blot Analysis

PFC tissues and treated cells were lysed in RIPA lysis buffer (Beyotime, Shanghai, China; Cat. No. P0013B) supplemented with a 1× general protease inhibitor cocktail (Beyotime; Cat. No. P1005; 100× stock diluted 1:100). The RIPA buffer contained 50 mM Tris (pH 7.4), 150 mM NaCl, 1% Triton X-100, 1% sodium deoxycholate, 0.1% SDS, and inhibitory components including sodium orthovanadate, sodium fluoride, EDTA, and leupeptin. Samples were homogenized and lysed at 4 °C, followed by centrifugation at 12,000× *g* for 15 min at 4 °C. The supernatants were collected, and total protein concentrations were determined using a Pierce™ BCA Protein Assay Kit (Thermo Fisher Scientific, Rockford, IL, USA) After denaturation, 25 μg of total protein from each sample was separated by SDS-PAGE and transferred onto BioTrace™ PVDF transfer membranes (Pall Corporation, Port Washington, NY, USA). Membranes were blocked with 5% non-fat milk for 1 h at room temperature and subsequently incubated overnight at 4 °C with the following primary antibodies diluted in blocking solution: CX3CL1 (Proteintech, Wuhan, China; Cat. No. 83738-5-RR, rabbit, 1:500), CX3CR1 (Proteintech, Wuhan, China; Cat. No. 66915-1-Ig, mouse, 1:2000), iNOS/NOS2 (Proteintech, Wuhan, China; Cat. No. 18985-1-AP, rabbit, 1:2000), Arginase-1 (Proteintech, Wuhan, China; Cat. No. 16001-1-AP, rabbit, 1:5000), β-actin (Proteintech, Wuhan, China; Cat. No. 66009-1-Ig, mouse, 1:20,000), and GAPDH (Proteintech, Wuhan, China; Cat. No. 60004-1-Ig, mouse, 1:50,000). Membranes were washed three times with TBST and then incubated for 1 h at room temperature with HRP-conjugated goat anti-rabbit IgG (Signalway Antibody, College Park, MD, USA; Cat. No. L3012, 1:5000) or HRP-conjugated goat anti-mouse IgG (Signalway Antibody; Cat. No. L3032, 1:10,000), as appropriate. Following three additional washes with TBST, immunoreactive bands were visualized using enhanced chemiluminescence. Images were acquired using a ChemiDoc MP Imaging System (Bio-Rad Laboratories, Inc., Hercules, CA, USA), and band intensities were quantified using ImageJ 1.53t and normalized to β-actin or GAPDH.

### 4.4. ELISA

IL-1β and IL-10 levels in PFC tissue homogenate supernatants and cell-culture supernatants were measured using commercial mouse sandwich ELISA kits (Shanghai Enzyme-linked Biotechnology Co., Ltd., Shanghai, China). The Mouse IL-1β ELISA Kit (Cat. No. ml106733) had a detection range of 23.44–1500 pg/mL and a sensitivity of 11.72 pg/mL, whereas the Mouse IL-10 ELISA Kit (Cat. No. ml037873) had a detection range of 7.81–500 pg/mL and a sensitivity of 3.91 pg/mL; according to the manufacturer, the sensitivity represents the minimum detectable concentration and was therefore regarded as the limit of detection. PFC tissues were homogenized in nine volumes of ice-cold PBS (1:9, *w*/*v*) and centrifuged at 12,000× *g* for 15 min at 4 °C. Total protein concentrations in tissue homogenate supernatants were determined using a BCA protein assay and were used for normalization of tissue cytokine levels. Cell-culture supernatants were centrifuged at 1000× *g* for 10 min at 4 °C. For ELISA, 100 μL of standards or samples was incubated at 37 °C for 1.5 h, followed by three washes, incubation with 100 μL biotinylated detection antibody at 37 °C for 1 h, four washes, and incubation with 100 μL streptavidin-HRP at 37 °C for 30 min. After four additional washes, 50 μL each of TMB substrate solutions A and B was added and incubated at 37 °C in the dark for 15 min. The reaction was stopped with 50 μL stop solution, absorbance was measured at 450 nm within 5 min, and cytokine concentrations were calculated from the corresponding standard curves; tissue cytokine levels were subsequently normalized to total protein content.

### 4.5. Immunofluorescence Staining

For tissue immunofluorescence, 5-μm paraffin sections were deparaffinized in xylene (2 × 10 min) and rehydrated through 100% ethanol (2 × 3 min), 95%, 85%, and 75% ethanol (2 min each), followed by distilled water for 2 min. Antigen retrieval was performed in EDTA buffer using a pressure cooker for 4 min after steam generation. Sections were permeabilized with 0.2% Triton X-100 for 10 min, washed with PBS (3 × 5 min), and blocked with 5% normal goat serum at 37 °C for 1 h. Primary antibodies were applied overnight at 4 °C: CX3CL1 (Proteintech, 83738-5-RR, 1:100), CX3CR1 (66915-1-Ig, 1:100), NeuN (Abcam, Cambridge, UK; Cat. No. ab104224, 1:500), and Iba1 (Proteintech, 10904-1-AP, 1:200). After PBS washing (3 × 5 min), sections were incubated with CoraLite594 goat anti-rabbit IgG (SA00013-4, 1:500) or CoraLite488 goat anti-mouse IgG (SA00013-1, 1:500) at 37 °C for 1 h in the dark. For AAV-transduced tissues, CoraLite647 goat anti-mouse IgG (SA00013-6, 1:400) replaced CoraLite488 when necessary to avoid overlap with viral green fluorescence; the 647 channel was pseudocolored for figure presentationSections were mounted with Antifade Mounting Medium with DAPI (Beyotime, Shanghai, China; Cat. No. P0131). Images were acquired using a Leica DMi8 microscope (Leica Microsystems, Wetzlar, Germany) under identical imaging settings between groups. Three sections per mouse, separated by five intervening sections, and three non-overlapping fields per section were analyzed. Mean fluorescence intensity was quantified using ImageJ 1.53t by an observer blinded to group allocation, with four mice per group.

For cell immunofluorescence, cells grown on coverslips were fixed with 4% paraformaldehyde for 20 min, washed with PBS (3 × 5 min), permeabilized with 0.1% Triton X-100 for 10 min, and washed twice with PBS (5 min each). After blocking with 5% normal goat serum for 60 min, cells were incubated overnight at 4 °C with CX3CL1 (83738-5-RR, 1:100), CX3CR1 (66915-1-Ig, 1:100), Arg1 (16001-1-AP, 1:200), or iNOS/NOS2 (18985-1-AP, 1:100). After PBS washing (3 × 5 min), cells were incubated with CoraLite594-conjugated goat anti-rabbit IgG (SA00013-4, 1:500) or CoraLite488-conjugated goat anti-mouse IgG (SA00013-1, 1:500) at 37 °C for 1 h in the dark. For lentivirally transduced cells, CoraLite647-conjugated goat anti-mouse IgG (SA00013-6, 1:400) was used instead of CoraLite488 to avoid overlap with the viral green fluorescence, whereas CoraLite594 was unchanged. The 647 fluorescence channel was pseudocolored as appropriate for figure presentation. Cells were mounted with Antifade Mounting Medium with DAPI (Beyotime, P0131). Images were acquired using a Leica DMi8 fluorescence microscope (Leica Microsystems, Wetzlar, Germany) with a 40× objective under identical imaging settings between groups. Five randomly selected, non-overlapping fields were analyzed per coverslip using ImageJ 1.53t by an observer blinded to group allocation, with four independent experiments performed per group.

Detailed information on the antibodies and ELISA kits used for Western blotting, immunofluorescence, and ELISA is summarized in Table 1.

### 4.6. Nissl Staining

Nissl staining was performed on 5-μm paraffin sections using cresyl violet solution (Solarbio, Beijing, China; Cat. No. G1430). Sections were deparaffinized in xylene (2 × 10 min) and rehydrated through 100% ethanol (2 × 3 min), 95%, 85%, and 75% ethanol (2 min each), followed by distilled water for 2 min. Sections were then stained with cresyl violet for 10 min at room temperature, differentiated in 95% ethanol until the background was clear, dehydrated, cleared, and mounted. Three sections per mouse were analyzed, with five intervening sections between analyzed sections, and three non-overlapping fields were evaluated per section. Quantification was performed within a predefined cortical region of the PFC using the same anatomical landmarks and the full cortical thickness across all groups; no separate layer- or subregion-specific analysis was performed. Neuronal integrity was calculated as the percentage of morphologically intact Nissl-positive neurons among total Nissl-positive neurons. Three mice were analyzed per group, and quantification was performed using ImageJ 1.53t by an observer blinded to group allocation.

### 4.7. TUNEL Staining

TUNEL staining was performed using a red-fluorescence apoptosis detection kit (KeyGEN BioTECH, Nanjing, China; Cat. No. KGA707). HT-22 cells were fixed with 4% paraformaldehyde for 20 min, washed with PBS (3 × 5 min), permeabilized with 0.1% Triton X-100 for 10 min, and washed again (2 × 5 min). The TUNEL reaction mixture was freshly prepared at a 1:9 ratio of TdT enzyme to labeling solution, and 50 μL was added per coverslip for incubation at 37 °C for 60 min in the dark. After washing (3 × 5 min), nuclei were counterstained with DAPI (1 μg/mL) for 5 min. Five randomly selected, non-overlapping fields were quantified per coverslip, with five independent experiments performed per group. Apoptosis was calculated as TUNEL-positive cells/total DAPI-positive cells × 100%.

### 4.8. Data Analysis

Investigators conducting the animal procedures were aware of group allocation, whereas quantitative image analysis was performed using ImageJ 1.53t by an observer blinded to group allocation. Western blot band intensities were normalized to the corresponding reference protein. Immunofluorescence images were acquired under identical exposure settings, and mean fluorescence intensity was measured within the target regions. Nissl and TUNEL staining were quantified using consistent thresholds, region-selection criteria, and analysis parameters across groups. Statistical analyses were performed using SPSS 26.0, and graphs were generated using GraphPad Prism v10.1.2. Data are presented as mean ± SD. Normality and homogeneity of variance were assessed using the Shapiro–Wilk and Levene tests, respectively. Comparisons between two groups were performed using independent-samples *t*-tests, whereas multiple-group comparisons were analyzed using one-way ANOVA followed by Tukey’s HSD post hoc test. When homogeneity of variance was not met, Tamhane’s T2 test was used for pairwise comparisons. *p* < 0.05 was considered statistically significant. Statistical significance is indicated in the figures as * *p* < 0.05, ** *p* < 0.01, and *** *p* < 0.001; ns indicates no significant difference.

## 5. Conclusions

This study showed that METH exposure was associated with reduced neuronal CX3CL1 expression, increased microglial CX3CR1 expression, neuroinflammatory changes, neuronal injury, and cognitive impairment in the PFC. Neuronal CX3CL1 overexpression and microglial CX3CR1 knockdown partially attenuated these changes, supporting the involvement of CX3CL1/CX3CR1 signaling in METH-associated PFC neuroinflammatory injury. Future studies should further characterize CX3CR1-associated intracellular signaling, distinguish the respective roles of membrane-bound and soluble CX3CL1, and determine whether similar CX3CL1/CX3CR1 alterations occur in the hippocampus and striatum.

## Figures and Tables

**Figure 1:**
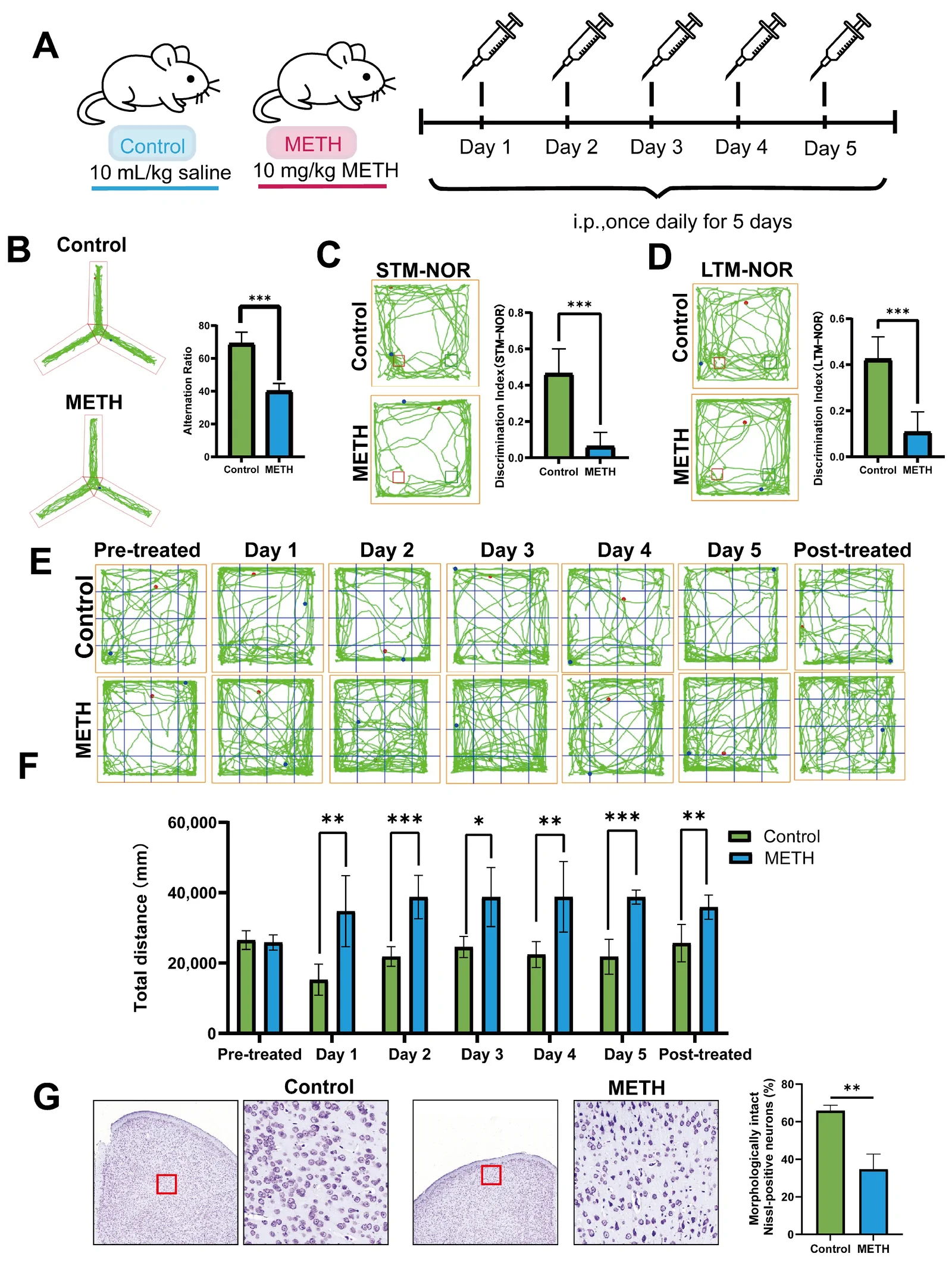
METH exposure induced cognitive deficits, hyperlocomotor activity, and neuronal injury in the PFC. (**A**) Experimental schematic of METH exposure and behavioral testing. (**B**) Representative movement trajectories in the Y-maze test and quantification of spontaneous alternation percentage. (**C**) Representative movement trajectories from the short-term memory novel object recognition test and quantification of the discrimination index. (**D**) Representative movement trajectories from the long-term memory novel object recognition test and quantification of the discrimination index. (**E**) Representative movement trajectories in the open-field test. (**F**) Quantification of total distance traveled before administration, during days 1–5 of METH treatment, and during the post-treatment testing phase. (**G**) Representative Nissl-stained images of the PFC and quantification of morphologically intact Nissl-positive neurons. Data are presented as mean ± SD. In (**B**–**E**), red and blue dots indicate the starting and ending positions of the animal trajectories, respectively. In (**C**,**D**), red and dark green boxes indicate the novel and familiar object zones, respectively. In (**E**), blue grid lines indicate the subdivision of the open-field arena. In (**G**), red boxes indicate the regions shown at higher magnification. Behavioral tests: *n* = 6 mice/group; Nissl staining: *n* = 3 mice/group. * *p* < 0.05, ** *p* < 0.01, *** *p* < 0.001.

**Figure 2:**
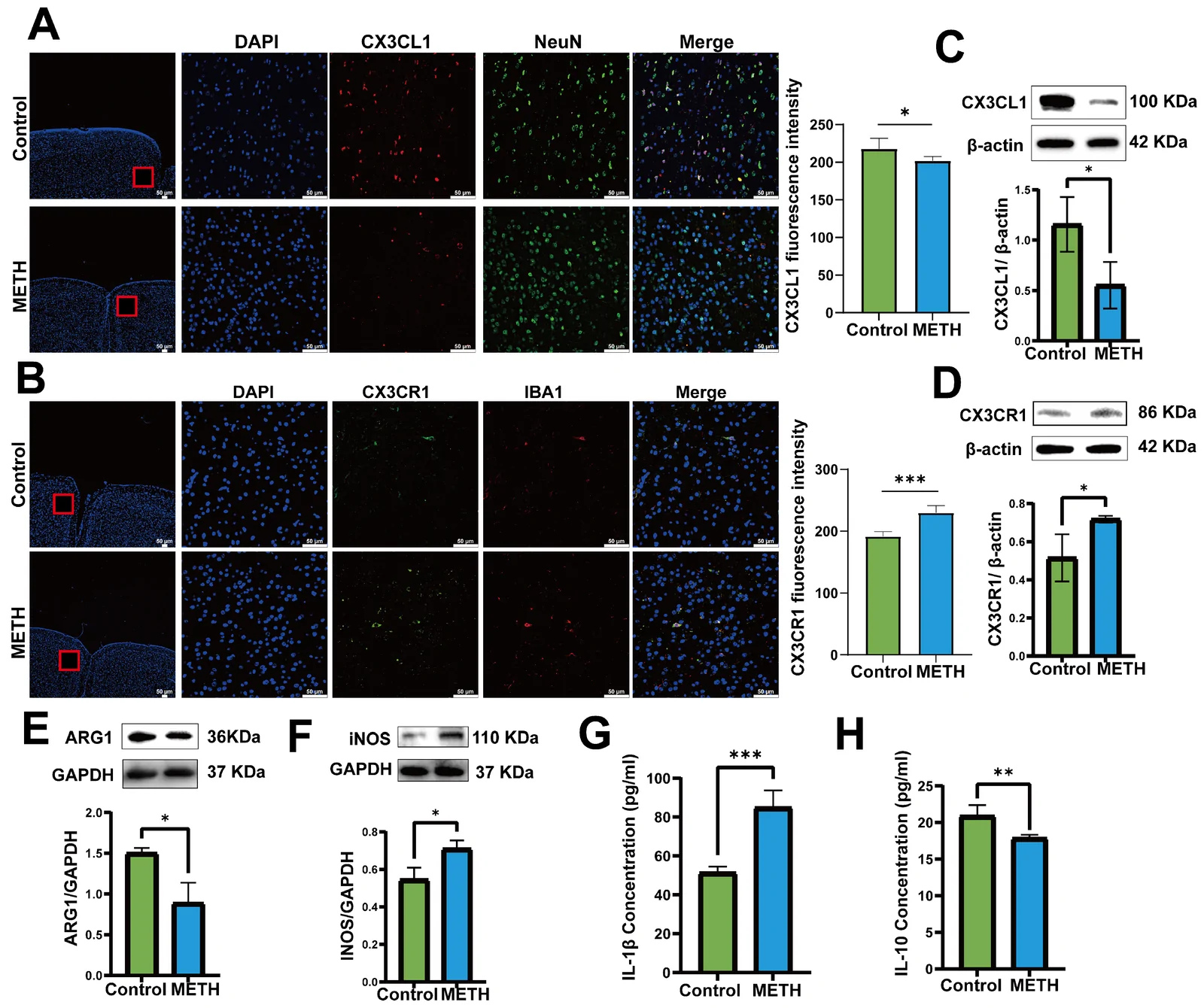
METH exposure disrupted CX3CL1/CX3CR1 signaling and promoted neuroinflammatory responses in the PFC. (**A**) Representative immunofluorescence co-staining images of CX3CL1 and the neuronal marker NeuN in the PFC, with quantification of CX3CL1 fluorescence intensity. (**B**) Representative immunofluorescence co-staining images of CX3CR1 and the microglial marker Iba1 in the PFC, with quantification of CX3CR1 fluorescence intensity. (**C**,**D**) Western blot analysis and densitometric quantification of CX3CL1 and CX3CR1 protein expression in the PFC. (**E**,**F**) Western blot analysis and densitometric quantification of Arg1 and iNOS protein expression in the PFC. (**G**,**H**) ELISA analysis of IL-1β and IL-10 levels in the PFC. Data are presented as mean ± SD. Red boxes in (**A**,**B**) indicate the regions shown at higher magnification. Immunofluorescence: *n* = 4 mice/group; Western blot analysis: *n* = 3 mice/group; ELISA: *n* = 5 mice/group. Scale bar = 50 μm. * *p* < 0.05, ** *p* < 0.01, *** *p* < 0.001.

**Figure 3:**
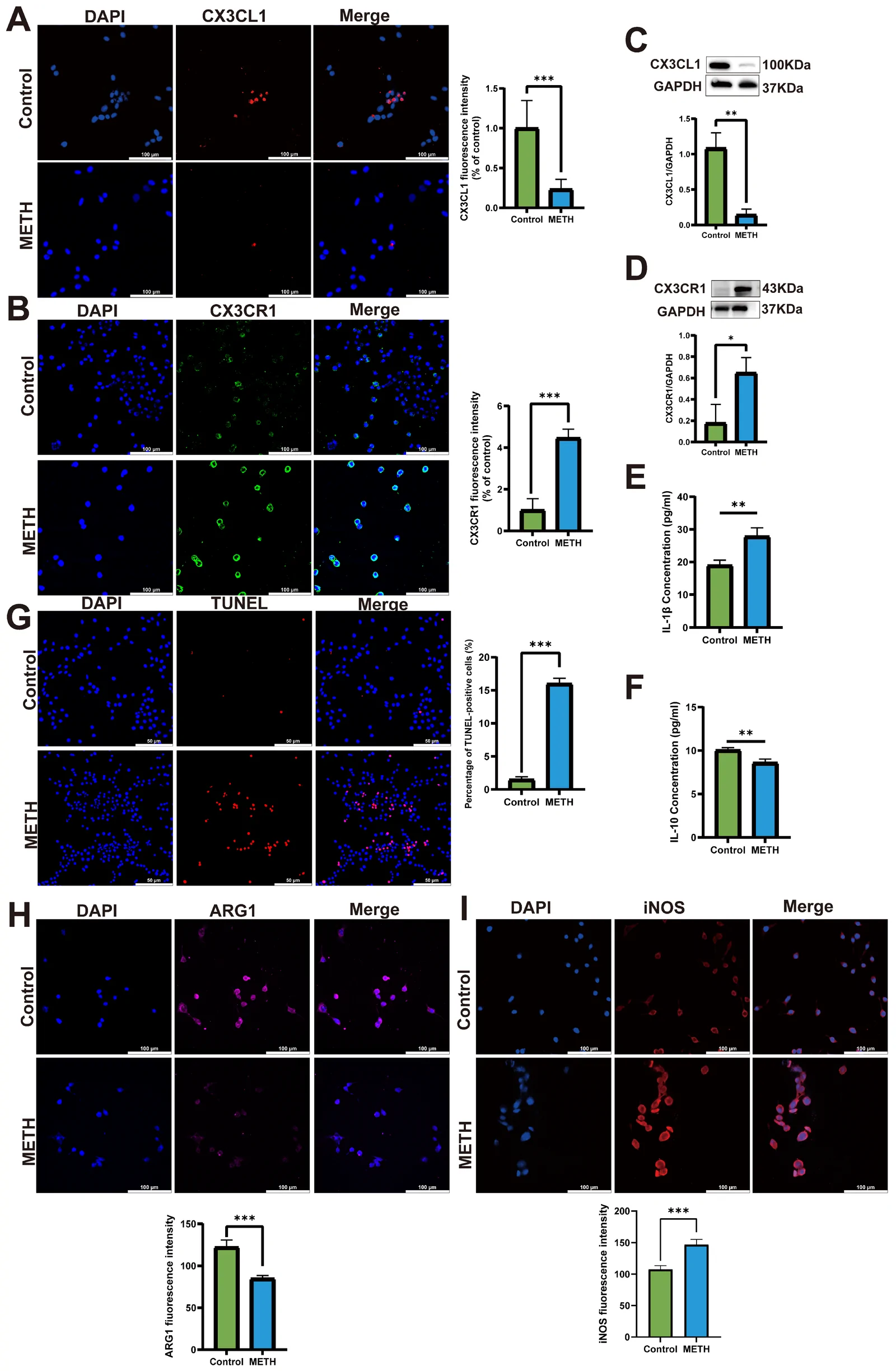
METH altered CX3CL1/CX3CR1 signaling and inflammatory phenotypes in HT-22 neuronal-like cells and BV2 microglia. (**A**) Representative immunofluorescence images of CX3CL1 in HT-22 neuronal-like cells and quantification of fluorescence intensity. (**B**) Representative immunofluorescence images of CX3CR1 in BV2 microglial cells and quantification of fluorescence intensity. (**C**,**D**) Western blot analysis and densitometric quantification of CX3CL1 protein expression in HT-22 neuronal-like cells and CX3CR1 protein expression in BV2 microglial cells. (**E**,**F**) ELISA analysis of IL-1β and IL-10 levels in cell culture supernatants. (**G**) Representative TUNEL staining of HT-22 neuronal-like cells and quantification of TUNEL-positive cells. (**H**,**I**) Representative immunofluorescence images of Arg1 and iNOS in BV2 microglial cells and quantification of fluorescence intensity. Data are presented as mean ± SD. Immunofluorescence: *n* = 4 independent experiments; Western blot analysis: *n* = 3 independent experiments; ELISA: *n* = 5 independent experiments; TUNEL: *n* = 5 independent experiments. Scale bar = 100 μm. * *p* < 0.05, ** *p* < 0.01, *** *p* < 0.001.

**Figure 4:**
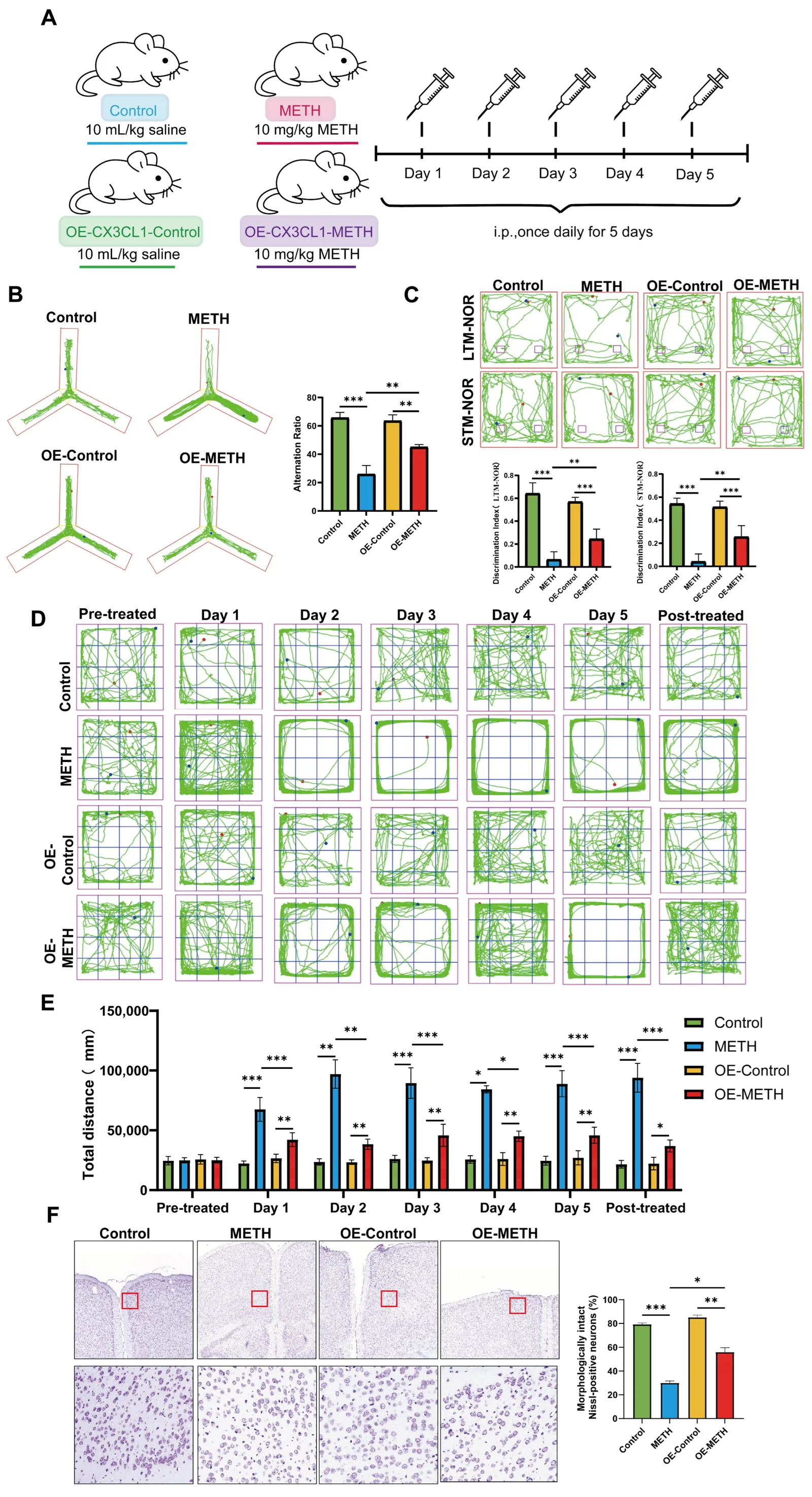
CX3CL1 overexpression attenuated METH-induced behavioral abnormalities and neuronal injury in the PFC. (**A**) Experimental schematic of AAV-mediated CX3CL1 overexpression, METH treatment, and behavioral testing. (**B**) Representative movement trajectories in the Y-maze test and quantification of spontaneous alternation percentage. (**C**) Representative movement trajectories from the short-term and long-term memory novel object recognition tests and quantification of the discrimination index. (**D**) Representative movement trajectories in the open-field test. (**E**) Quantification of total distance traveled before administration, during days 1–5 of METH treatment, and during the post-treatment testing phase. (**F**) Representative Nissl-stained images of the PFC and quantification of morphologically intact Nissl-positive neurons. Data are presented as mean ± SD. In (**B**–**D**), red and blue dots indicate the starting and ending positions of the animal trajectories, respectively. In (**C**), red and dark green boxes indicate the novel and familiar object zones, respectively. In (**D**), blue grid lines indicate the subdivision of the open-field arena. In (**F**), red boxes indicate the regions shown at higher magnification. Behavioral tests: *n* = 6 mice/group; Nissl staining: *n* = 3 mice/group. * *p* < 0.05, ** *p* < 0.01, *** *p* < 0.001.

**Figure 5:**
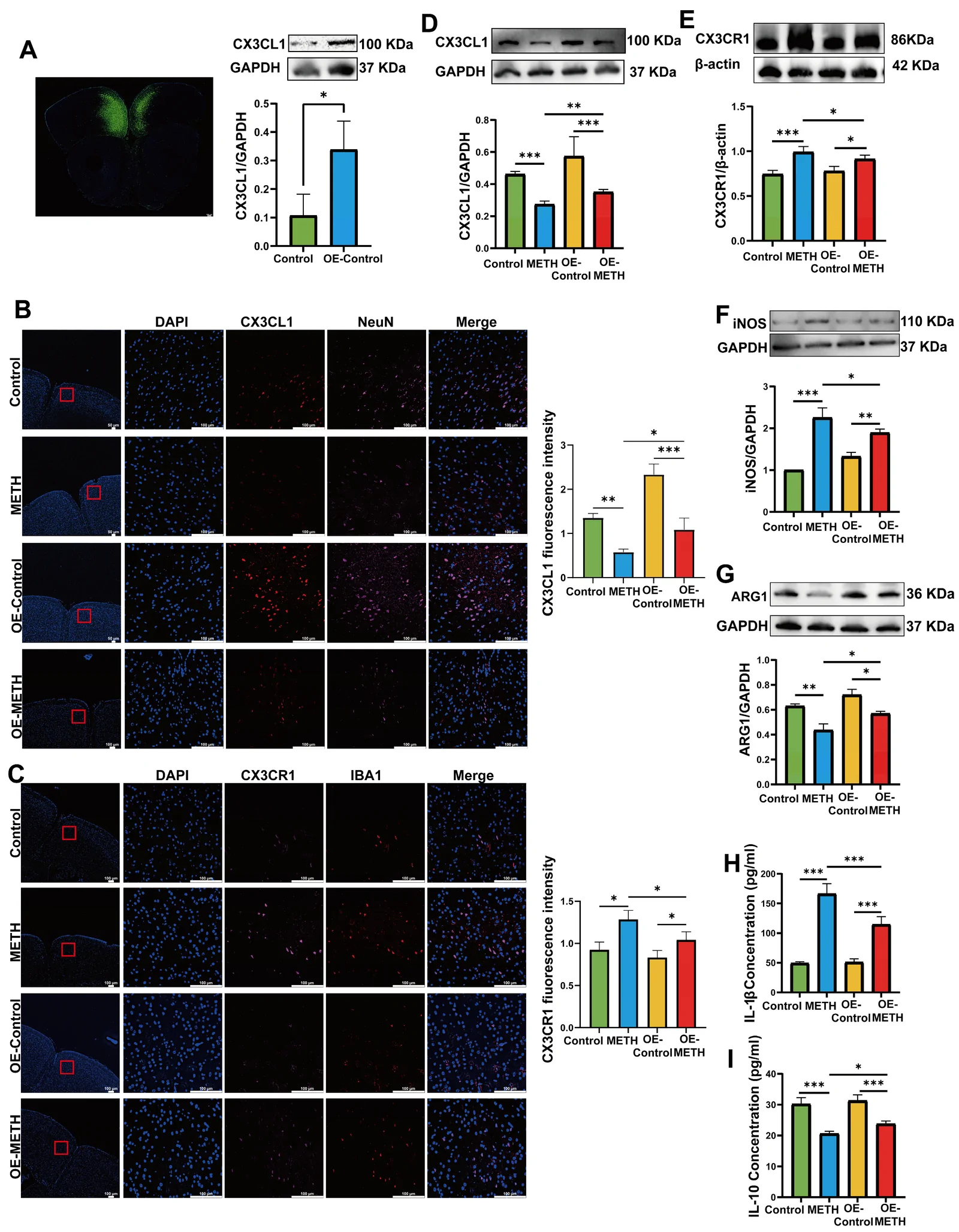
CX3CL1 overexpression attenuated METH-induced CX3CL1/CX3CR1 axis dysregulation and neuroinflammatory responses in the PFC. (**A**) Representative images of AAV-CX3CL1 expression and Western blot validation of CX3CL1 overexpression efficiency. (**B**) Representative immunofluorescence co-staining images of CX3CL1 and NeuN in the PFC, with quantification of CX3CL1 fluorescence intensity. (**C**) Representative immunofluorescence co-staining images of CX3CR1 and Iba1 in the PFC, with quantification of CX3CR1 fluorescence intensity. (**D**,**E**) Western blot analysis and densitometric quantification of CX3CL1 and CX3CR1 protein expression in the PFC. (**F**,**G**) Western blot analysis and densitometric quantification of iNOS and Arg1 protein expression in the PFC. For the four-lane Western blots in panels (**D**–**G**), lanes from left to right correspond to Control, METH, OE-Control, and OE-METH. (**H**,**I**) ELISA analysis of IL-1β and IL-10 levels in the PFC. Data are presented as mean ± SD. Red boxes in (**B**,**C**) indicate the regions shown at higher magnification. Immunofluorescence: *n* = 4 mice/group; Western blot analysis: *n* = 3 mice/group; ELISA: *n* = 5 mice/group. Scale bar = 100 μm. * *p* < 0.05, ** *p* < 0.01, *** *p* < 0.001.

**Figure 6:**
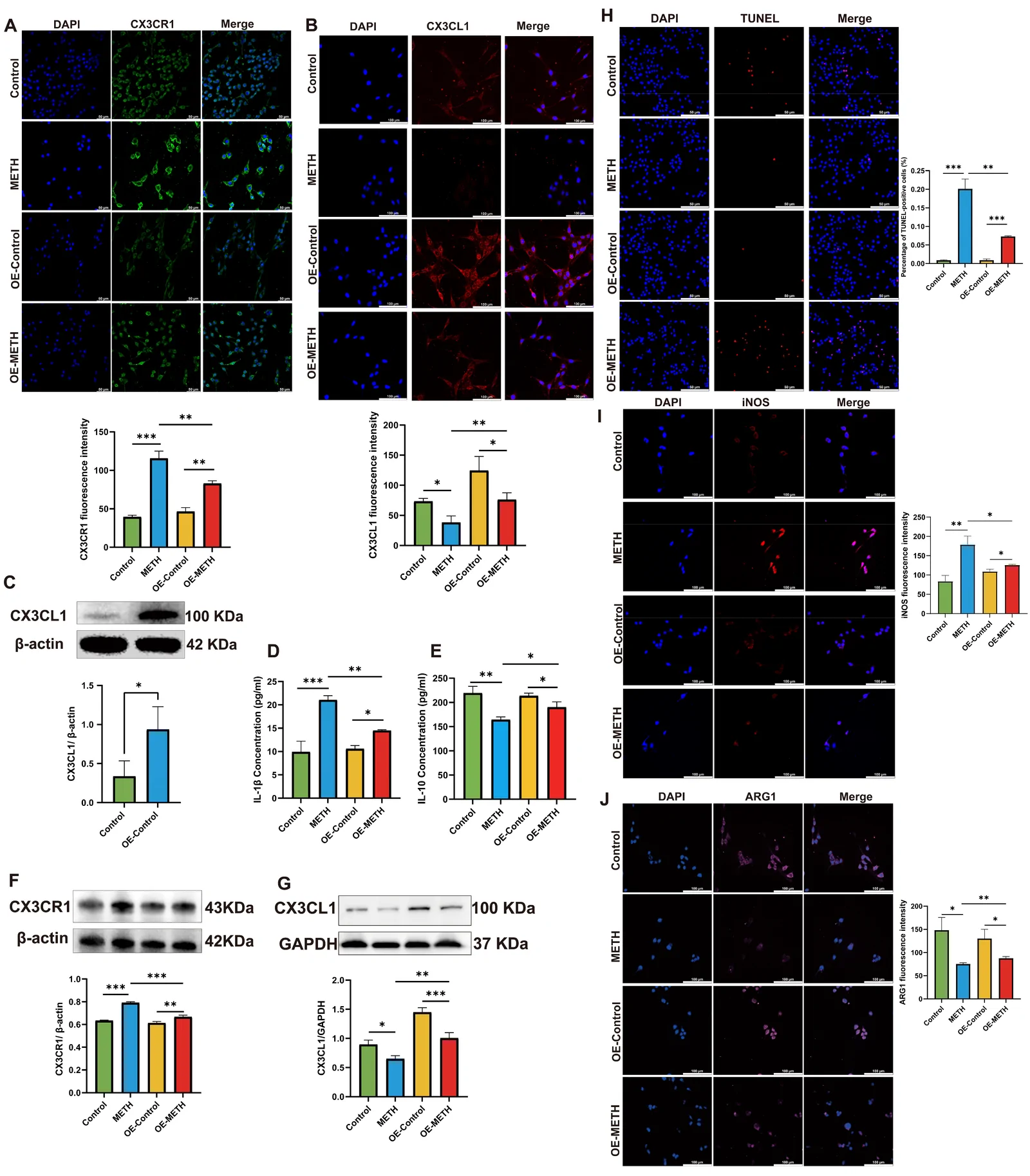
CX3CL1 overexpression attenuated METH-induced neuronal injury and microglial inflammatory responses in the HT-22/BV2 coculture system. (**A**) Representative immunofluorescence images of CX3CR1 in the coculture system and quantification of fluorescence intensity. (**B**) Representative immunofluorescence images of CX3CL1 in the coculture system and quantification of fluorescence intensity. (**C**) Western blot validation of CX3CL1 overexpression efficiency in HT-22 neuronal-like cells. (**D**,**E**) ELISA analysis of IL-1β and IL-10 levels in coculture supernatants. (**F**,**G**) Western blot analysis and densitometric quantification of CX3CR1 and CX3CL1 protein expression in the coculture system. For the four-lane Western blots in panels (**F**,**G**) lanes from left to right correspond to Control, METH, OE-Control, and OE-METH. (**H**) Representative TUNEL staining of HT-22 neuronal-like cells and quantification of TUNEL-positive cells. (**I**,**J**) Representative immunofluorescence images of iNOS and Arg1 in BV2 microglial cells and quantification of fluorescence intensity. Data are presented as mean ± SD. Immunofluorescence: *n* = 4 independent experiments; Western blot analysis: *n* = 3 independent experiments; ELISA: *n* = 5 independent experiments; TUNEL: *n* = 5 independent experiments. Scale bar = 100 μm. * *p* < 0.05, ** *p* < 0.01, *** *p* < 0.001.

**Figure 7:**
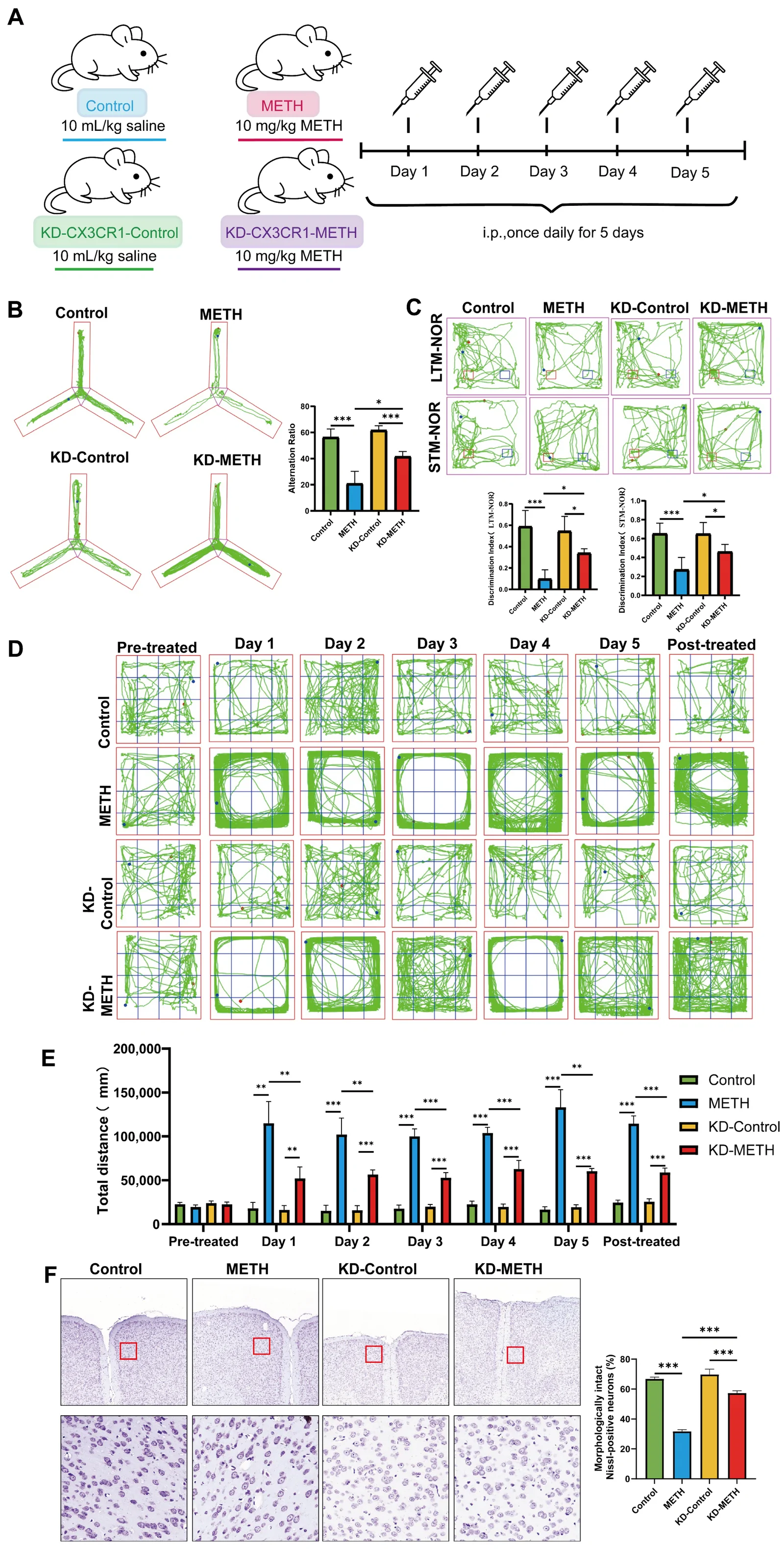
CX3CR1 knockdown attenuated METH-induced behavioral abnormalities and neuronal injury in the PFC. (**A**) Experimental schematic of AAV-mediated CX3CR1 knockdown, METH treatment, and behavioral testing. (**B**) Representative movement trajectories in the Y-maze test and quantification of spontaneous alternation percentage. (**C**) Representative movement trajectories from the short-term and long-term memory novel object recognition tests and quantification of the discrimination index. (**D**) Representative movement trajectories in the open-field test. (**E**) Quantification of total distance traveled before administration, during days 1–5 of METH treatment, and during the post-treatment testing phase. (**F**) Representative Nissl-stained images of the PFC and quantification of morphologically intact Nissl-positive neurons. Data are presented as mean ± SD. In (**B**–**D**), red and blue dots indicate the starting and ending positions of the animal trajectories, respectively. In (**C**), red and dark green boxes indicate the novel and familiar object zones, respectively. In (**D**), blue grid lines indicate the subdivision of the open-field arena. In (**F**), red boxes indicate the regions shown at higher magnification.Behavioral tests: *n* = 6 mice/group; Nissl staining: *n* = 3 mice/group. * *p* < 0.05, ** *p* < 0.01, *** *p* < 0.001.

**Figure 8:**
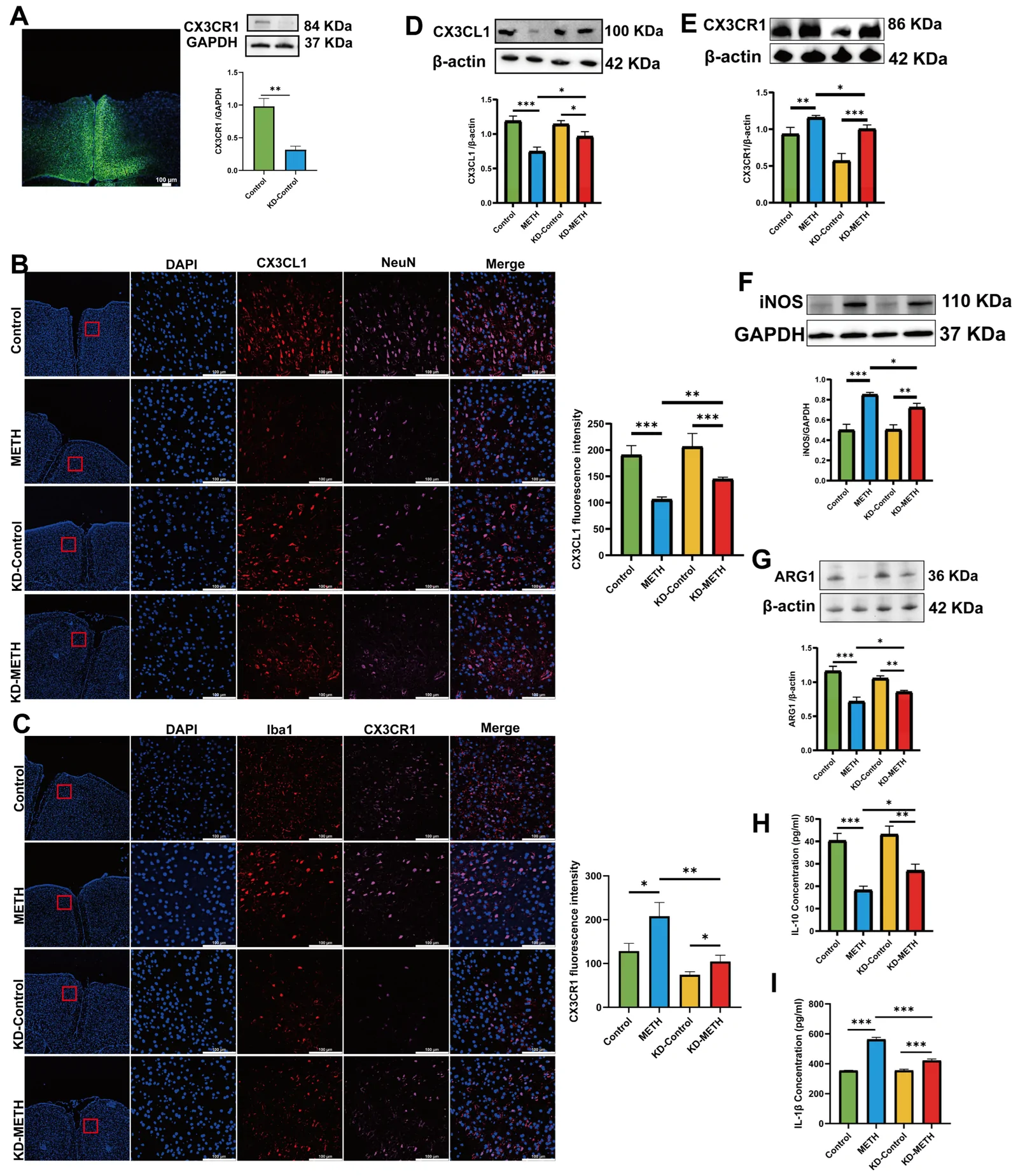
CX3CR1 knockdown attenuated METH-induced CX3CL1/CX3CR1 axis dysregulation and neuroinflammatory responses in the PFC. (**A**) Representative images of AAV-shCX3CR1 expression and Western blot validation of CX3CR1 knockdown efficiency. (**B**) Representative immunofluorescence co-staining images of CX3CL1 and NeuN in the PFC, with quantification of CX3CL1 fluorescence intensity. (**C**) Representative immunofluorescence co-staining images of CX3CR1 and Iba1 in the PFC, with quantification of CX3CR1 fluorescence intensity. (**D**,**E**) Western blot analysis and densitometric quantification of CX3CL1 and CX3CR1 protein expression in the PFC. (**F**,**G**) Western blot analysis and densitometric quantification of iNOS and Arg1 protein expression in the PFC. For the four-lane Western blots in panels (**D**–**G**), lanes from left to right correspond to Control, METH, KD-Control, and KD-METH. (**H**,**I**) ELISA analysis of IL-10 and IL-1β levels in the PFC. Data are presented as mean ± SD. Immunofluorescence: *n* = 4 mice/group; Western blot analysis: *n* = 3 mice/group; ELISA: *n* = 5 mice/group. Scale bar = 100 μm. * *p* < 0.05, ** *p* < 0.01, *** *p* < 0.001.

**Figure 9:**
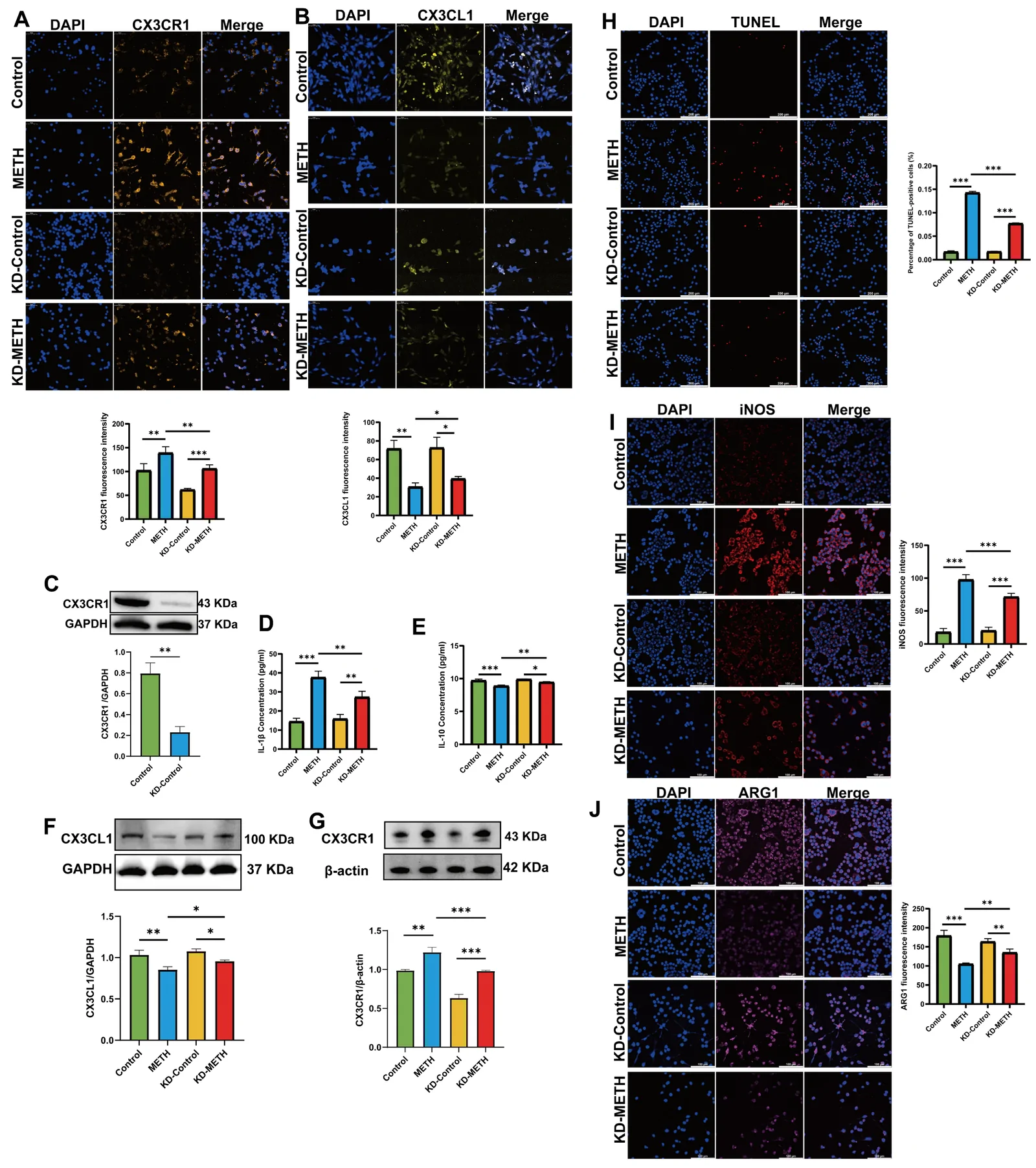
CX3CR1 knockdown attenuated METH-induced neuronal injury and microglial inflammatory responses in the HT-22/BV2 coculture system. (**A**) Representative immunofluorescence images of CX3CR1 in the coculture system and quantification of fluorescence intensity. (**B**) Representative immunofluorescence images of CX3CL1 in the coculture system and quantification of fluorescence intensity. (**C**) Western blot validation of CX3CR1 knockdown efficiency in BV2 microglial cells. (**D**,**E**) ELISA analysis of IL-1β and IL-10 levels in coculture supernatants. (**F**,**G**) Western blot analysis and densitometric quantification of CX3CL1 and CX3CR1 protein expression in the coculture system. For the four-lane Western blots in panels (**F**,**G**), lanes from left to right correspond to Control, METH, KD-Control, and KD-METH. (**H**) Representative TUNEL staining of HT-22 neuronal-like cells and quantification of TUNEL-positive cells. (**I**,**J**) Representative immunofluorescence images of iNOS and Arg1 in BV2 microglial cells and quantification of fluorescence intensity. Data are presented as mean ± SD. Immunofluorescence: *n* = 4 independent experiments; Western blot analysis: *n* = 3 independent experiments; ELISA: *n* = 5 independent experiments; TUNEL: *n* = 5 independent experiments. Scale bar = 100 μm. * *p* < 0.05, ** *p* < 0.01, *** *p* < 0.001.

**Table 1:** Antibodies and commercial kits used in this study.

Technique	Reagent Type	Target/Reagent	Manufacturer	Catalog No.	Dilution/Detection Range	Incubation/Application
Western blot	Primary antibody	CX3CL1	Proteintech, Wuhan, China	83738-5-RR	1:500	Overnight at 4 °C
Western blot	Primary antibody	CX3CR1	Proteintech, Wuhan, China	66915-1-Ig	1:2000	Overnight at 4 °C
Western blot	Primary antibody	iNOS/NOS2	Proteintech, Wuhan, China	18985-1-AP	1:2000	Overnight at 4 °C
Western blot	Primary antibody	Arginase-1 (Arg1)	Proteintech, Wuhan, China	16001-1-AP	1:5000	Overnight at 4 °C
Western blot	Primary antibody	β-actin	Proteintech, Wuhan, China	66009-1-Ig	1:20,000	Overnight at 4 °C
Western blot	Primary antibody	GAPDH	Proteintech, Wuhan, China	60004-1-Ig	1:50,000	Overnight at 4 °C
Western blot	Secondary antibody	HRP-conjugated goat anti-rabbit IgG	Signalway Antibody, College Park, MD, USA	L3012	1:5000	1 h at room temperature
Western blot	Secondary antibody	HRP-conjugated goat anti-mouse IgG	Signalway Antibody, College Park, MD, USA	L3032	1:10,000	1 h at room temperature
Immunofluorescence	Primary antibody	CX3CL1	Proteintech, Wuhan, China	83738-5-RR	1:100	Overnight at 4 °C; tissue and cells
Immunofluorescence	Primary antibody	CX3CR1	Proteintech, Wuhan, China	66915-1-Ig	1:100	Overnight at 4 °C; tissue and cells
Immunofluorescence	Primary antibody	NeuN	Abcam, Cambridge, UK	ab104224	1:500	Overnight at 4 °C; tissue
Immunofluorescence	Primary antibody	Iba1	Proteintech, Wuhan, China	10904-1-AP	1:200	Overnight at 4 °C; tissue
Immunofluorescence	Primary antibody	Arginase-1 (Arg1)	Proteintech, Wuhan, China	16001-1-AP	1:200	Overnight at 4 °C; cells
Immunofluorescence	Primary antibody	iNOS/NOS2	Proteintech, Wuhan, China	18985-1-AP	1:100	Overnight at 4 °C; cells
Immunofluorescence	Secondary antibody	CoraLite594 goat anti-rabbit IgG	Proteintech, Wuhan, China	SA00013-4	1:500	1 h at 37 °C in the dark
Immunofluorescence	Secondary antibody	CoraLite488 goat anti-mouse IgG	Proteintech, Wuhan, China	SA00013-1	1:500	1 h at 37 °C in the dark
Immunofluorescence	Secondary antibody	CoraLite647 goat anti-mouse IgG	Proteintech, Wuhan, China	SA00013-6	1:400	1 h at 37 °C in the dark; used when required to avoid overlap with viral green fluorescence
ELISA	Commercial kit	Mouse IL-1β ELISA Kit	Shanghai Enzyme-linked Biotechnology Co., Ltd., Shanghai, China	ml106733	23.44–1500 pg/mL; sensitivity 11.72 pg/mL	Sandwich ELISA
ELISA	Commercial kit	Mouse IL-10 ELISA Kit	Shanghai Enzyme-linked Biotechnology Co., Ltd., Shanghai, China	ml037873	7.81–500 pg/mL; sensitivity 3.91 pg/mL	Sandwich ELISA

**Abbreviations:** ELISA, enzyme-linked immunosorbent assay; HRP, horseradish peroxidase; iNOS/NOS2, inducible nitric oxide synthase; Arg1, arginase-1. For ELISA kits, sensitivity was regarded as the limit of detection according to the manufacturer. CoraLite647-conjugated secondary antibody was used when necessary to avoid spectral overlap with viral green fluorescence.

## Data Availability

The data generated and analyzed in this study are available from the corresponding author upon reasonable request.
